# Self-delivering RNAi compounds as therapeutic agents in the central nervous system to enhance axonal regeneration after injury

**DOI:** 10.1016/j.isci.2022.104379

**Published:** 2022-05-10

**Authors:** Sarah A. Woller, Joerg Ruschel, Barbara Morquette, James Cardia, Dinxue Yan, Katherine Holton, Taisia Shmushkovich, Emily Niederst, Karen Bulock, Alexey Wolfson, Matthew Abbinanti, Alyson E. Fournier, Lisa McKerracher, Kenneth M. Rosen

**Affiliations:** 1 BioAxone BioSciences Inc., Boston, MA, USA; 2 Montréal Neurological Institute, McGill University, Montréal, QC, Canada; 3 Phio Pharmaceuticals, Marlborough, MA, USA; 4 Advirna, LLC, Worcester, MA, USA

**Keywords:** Biotechnology, Health sciences, Techniques in neuroscience

## Abstract

The therapeutic use of RNAi has grown but often faces several hurdles related to delivery systems, compound stability, immune activation, and on-target/off-tissue effects. Self-delivering RNAi (sdRNA) molecules do not require delivery agents or excipients. Here we demonstrate the ability of sdRNA to reduce the expression of PTEN (phosphatase and tensin homolog) to stimulate regenerative axon regrowth in the injured adult CNS. PTEN-targeting sdRNA compounds were tested for efficacy *in vivo* by intravitreal injection after adult rat optic nerve injury. We describe critical steps in lead compound generation through the optimization of nucleotide modifications, enhancements for stability in biological matrices, and screening for off-target immunostimulatory activity. The data show that PTEN expression *in vivo* can be reduced using sdRNA and this enhances regeneration in adult CNS neurons after injury, raising the possibility that this method could be utilized for other clinically relevant nervous system indications.

## Highlights

• self-delivering siRNA (sdRNA) can decrease neuronal gene expression *in vivo*
• sdRNA can be successfully delivered *in vivo* without using vectors or excipients
• Phosphatase and tensin homolog (PTEN)-targeting sdRNA can enhance CNS neuronal regeneration after injury

## Graphical abstract

**Figure:**
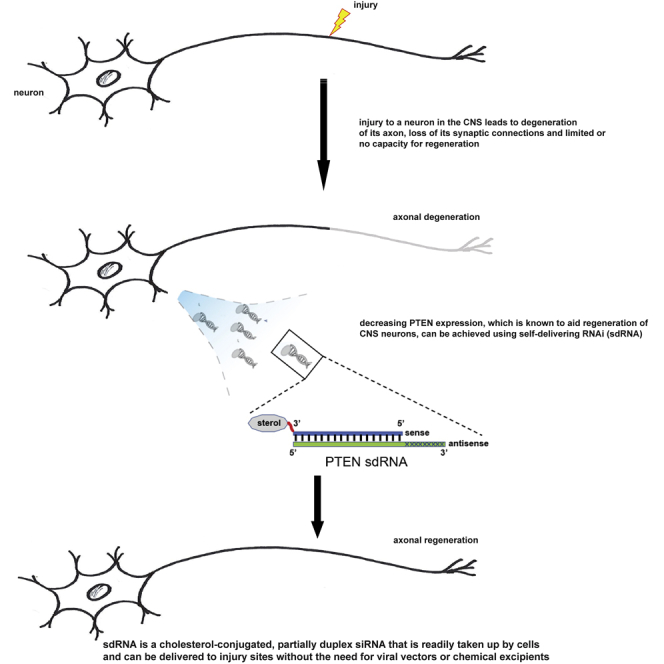

## Introduction

RNAinterference (RNAi) is a pathway by which cells can use homologous antisense RNA sequences to anneal with and direct the degradation of targeted RNA molecules. The use of this technology for therapeutic applications, where post-transcriptional control of mRNA can be leveraged to reduce the production of specific proteins has grown dramatically over the last few years. RNAi compounds silence gene expression in mammalian cells by binding to a homologous mRNA sequence to elicit its degradation via the RNA-induced silencing complex, or RISC, preventing protein translation. RNAi compounds show their greatest potential when targeting mRNAs whose corresponding proteins are either inaccessible or incapable of being targeted with small molecules or other typical therapeutics. Although there have been problems historically associated with the delivery and stability of RNAi compounds, some of these issues have been solved and several RNAi drugs are presently approved or in late-stage clinical trials (Kaczmarek et al., 2017; Kuehn, 2018).

Self-delivering RNAi’s (sdRNA) are RNA duplexes where one strand is conjugated to a cholesteryl moiety through a triethylene glycol linker to allow efficient, direct cell membrane penetration (Ly et al., 2017) without the need for special delivery carriers or excipients including liposomes, nanoparticles or others. The nucleic acid region is an asymmetric duplex containing a 19–23 base long guide strand (antisense strand) and a passenger strand (sense strand) of 11–15 bases. Both strands have additional chemical modifications to confer nuclease stability, reduce net charge, enhance hydrophobicity, and limit immune-stimulating behavior (Byrne et al., 2013; Shmushkovich et al., 2018). The duplex region typically contains additional 2′OMe and 2′F internal backbone modifications and all nucleotides in the single-stranded overhang have fully phosphorothioylated ester linkages. The combination of features allows sdRNA compounds to achieve efficient spontaneous cellular uptake and potent gene silencing (Alterman et al., 2015; Byrne et al., 2013; Ly et al., 2017). Critically, access to the CNS for many therapeutics is rendered much more difficult by the behavior and function of the blood–brain barrier, limiting the ability to use peripherally delivered therapeutics, especially large molecule biologic drugs, in treating CNS indications. And while viral vector technologies have improved significantly over the last 10 or more years, they still face significant hurdles when employed for use in the CNS. An understanding of RNAi compounds as drugs (Kuehn, 2018), together with this recent sdRNA technology, prompted us to investigate the potential of this method to modify the response of adult central neurons after they have been injured, a notoriously difficult problem. To test the capacity of sdRNA to target pro-regenerative signaling pathways in CNS neurons, we chose to create sdRNA molecules to block the expression of phosphatase and tensin homolog (PTEN).

Neurons in the adult CNS fail to regenerate their axons after injury, which results in a loss of neuronal connections and often serious and permanent neurological deficits, such as paralysis after spinal cord injury (SCI). Major advances have been made in the identification of signaling pathways to promote axon regeneration, although there are no approved drugs to foster regenerative repair after neurotrauma. Axon regeneration in the CNS is blocked by both extrinsic and intrinsic barriers to regeneration, and robust axon regeneration fails even when CNS damage and scar formation are minimal (Canty et al., 2013; Erturk et al., 2007; Kerschensteiner et al., 2005). Intrinsic barriers to regeneration encompass the decline in the innate regenerative potential of adult neurons compared to young or developing neurons (Blackmore and Letourneau, 2006; Daugherty and Raz, 2015; Goldberg, 2004; Hilton and Bradke, 2017). Drugs that “awaken” neurons to regenerate have great potential to help repair the injured CNS. Of the targets that address the intrinsic barriers to regeneration, PTEN has been perhaps the most promising although results are more robust in experiments with transgenic mice than in rats treated with shRNA, perhaps because of the differences in the length of the optic nerve as well as technical differences in treatment protocols (Du et al., 2015; Geoffroy et al., 2016; He and Jin, 2016; Lewandowski and Steward, 2014; Liu et al., 2010; Park et al., 2008; Zukor et al., 2013). PTEN is a phosphatase that dephosphorylates phosphatidylinositol 3-phosphates and negatively regulates phosphatidylinositol 3-kinase (PI3K) and Akt (protein kinase B). PTEN also indirectly inhibits the activity of the mammalian target of rapamycin (mTOR) (He and Jin, 2016). Permanent PTEN gene knockout in motor cortex using viral delivery promotes axon regeneration in mice and rats after acute (Lewandowski and Steward, 2014; Liu et al., 2010; Zukor et al., 2013) and chronic (Du et al., 2015) SCI. Although these studies validate PTEN gene silencing as a promising strategy to overcome intrinsic barriers to axon regeneration in the CNS, drugs that target PTEN in a specific and therapeutically relevant fashion have not been developed.

Here we describe the screening and testing of specific sdRNA molecules to create a potential therapeutic to knock down PTEN expression in the CNS to promote axon regeneration after injury. We identified a PTEN targeting sequence that mediated efficient knockdown of PTEN levels and with sequence homology to rat, pig, primate, and human PTEN mRNA to facilitate further translational drug development. We used the adult rat optic nerve to test the *in vivo* performance of our drug candidate because adult rat retinal ganglion cells (RGC) are quite refractory to spontaneous regeneration. Also with no collateral branches, regeneration is not confused with sprouting from uninjured fibers. Looking beyond PTEN silencing, creating sdRNA drugs that can be delivered by injection into the CNS without the need for additional vectors or excipients or formulation holds promise for the treatment of an array of neurotraumas and neurological diseases.

## Results

### Primary screening with rat and human cell lines

**Figure 1:**
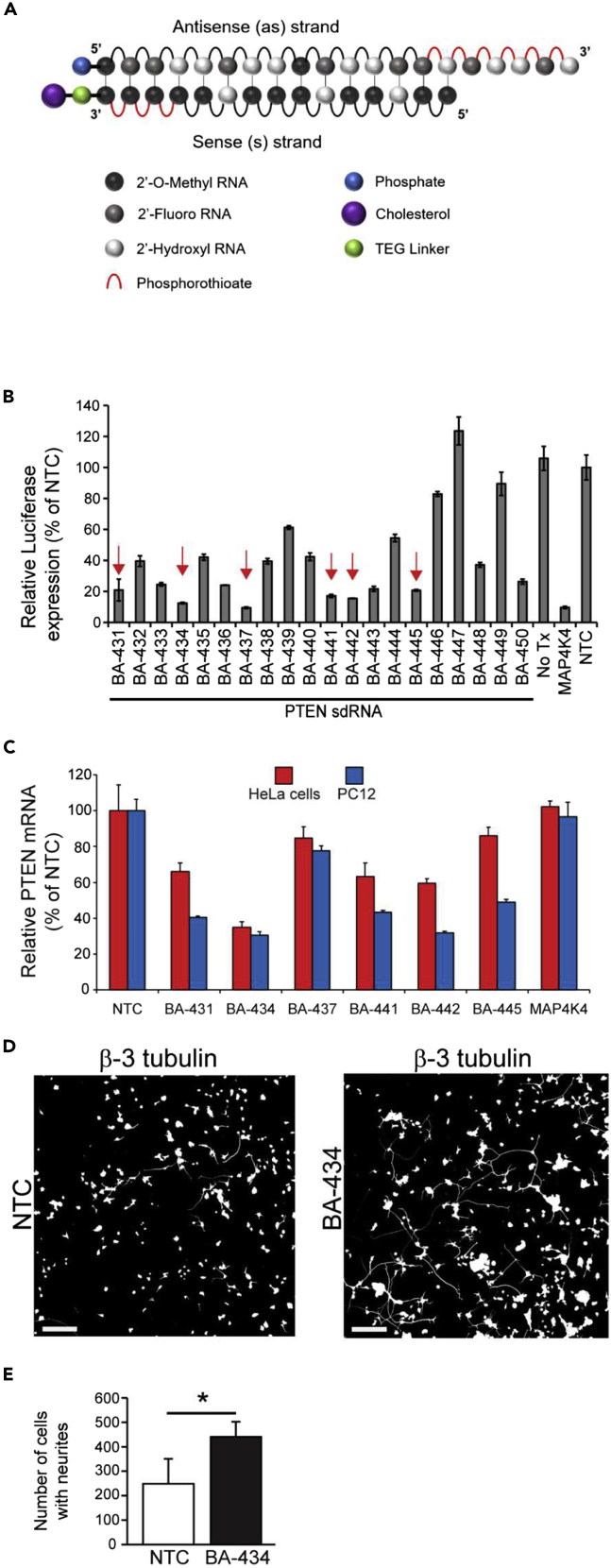
Structure and screening of PTEN sdRNAs (A) Representative schematic structure of a self-delivering siRNA (sdRNA) consisting of asymmetric siRNA duplexes with antisense overhang and chemically modified ribonucleotides conjugated to a cholesterol ester. (B) HeLa cells transfected with PTEN-luciferase reporter plasmid and treated with 2 μM PTEN sdRNA, or construct and sdRNA for MAP4K4 as a positive control or NTC (non-targeting control) sdRNAs or left untreated (NoTx) negative controls. The different sdRNA sequences tested are numbered. Normalized luciferase expression is plotted as a percentage of NTC treated cells. (C) Relative PTEN mRNA expression in HeLa cells (red) and PC-12 cells (blue) treated with 2 μM PTEN, MAP4K4, or NTC sdRNAs for 48 h. The different sdRNA sequences tested are numbered. (D) Representative images of PC-12 cells 4 days after treatment with 1 μM non-targeting control (NTC) or BA-434 and stained for visualizing neurite outgrowth using anti-β3 tubulin. Scale bar = 100 μm. (E) Total number of cells with one or more neurites, plotted as mean +SEM ∗p < 0.05 by Student’s *t* test.

The chemical modifications associated with sdRNA sequences (Figure 1A) restrict the sequence space for effective mRNA silencing compared to traditional siRNAs (Khvorova and Watts, 2017; Shmushkovich et al., 2018). An *in silico* algorithm was used to predict functional rat sdRNA sequences (Shmushkovich et al., 2018), and we screened 20 different duplexes that targeted PTEN (Table S1) to increase the probability of selecting a suitable sdRNA, preferably recognizing other species and enhancing the prospect of drug translatability. Reactivity against both humans and rats is important as both efficacy and safety studies to be performed on rats would use a PTEN sequence identical to that of humans.

Double-stranded sdRNA molecules were prepared by annealing the selected guide and passenger strands and duplex formation was confirmed using native gel electrophoresis (see Figure S1A). Screening of the 20 potential PTEN-targeting sdRNA sequences using a luciferase reporter system identified 18 compounds that reduced Renilla luciferase expression by at least 20% as compared to a non-targeting control (NTC) sdRNA (Figure 1B). Of these, we selected the six most efficient sdRNAs (Figure 1B, arrows) to test in human HeLa cells and rat PC-12 cells. qPCR (Q-PCR) assays established which of the six candidates significantly reduced PTEN mRNA levels in both HeLa and PC-12 cells after being delivered simply by direct addition to the culture medium (Figure 1C). A dose-response experiment (see Figure S1B) confirmed BA-434, BA-441, and BA-442 showed the greatest potency. Ultimately, BA-434 was selected as the lead because it showed the strongest reduction of PTEN mRNA, with 1 μM causing a 75% reduction in HeLa cells, while those treated with BA-441 or BA-442 showed only a 60% decrease (see Figure S1B). BA-434 was also the best at reducing PTEN mRNA (>60% reduction) in PC-12 cells (Figure 1C). Importantly, as stated above, the targeting sequence of BA-434 is identical between rats and humans.

We next evaluated whether direct media application of BA-434 could promote neurite outgrowth, serving as a physiologically relevant output for proof-of-concept, using PC-12 cells as a test platform. PC-12 cells were cultured in a medium with sub-optimal concentrations of NGF, limiting neurite outgrowth, and were then treated by the addition to the media of either BA-434 or NTC (non-targeting control) sdRNA. Neurites were visualized by immunostaining with βIII tubulin (Figure 1D) and quantitative analysis (Figure 1E) showed that suppression of PTEN expression by BA-434 treatment significantly increased the number of cells with neurites (T-test, p < 0.05).

### Primary neurons take up BA-434 and have sustained knockdown of phosphatase and tensin homolog protein

**Figure 2:**
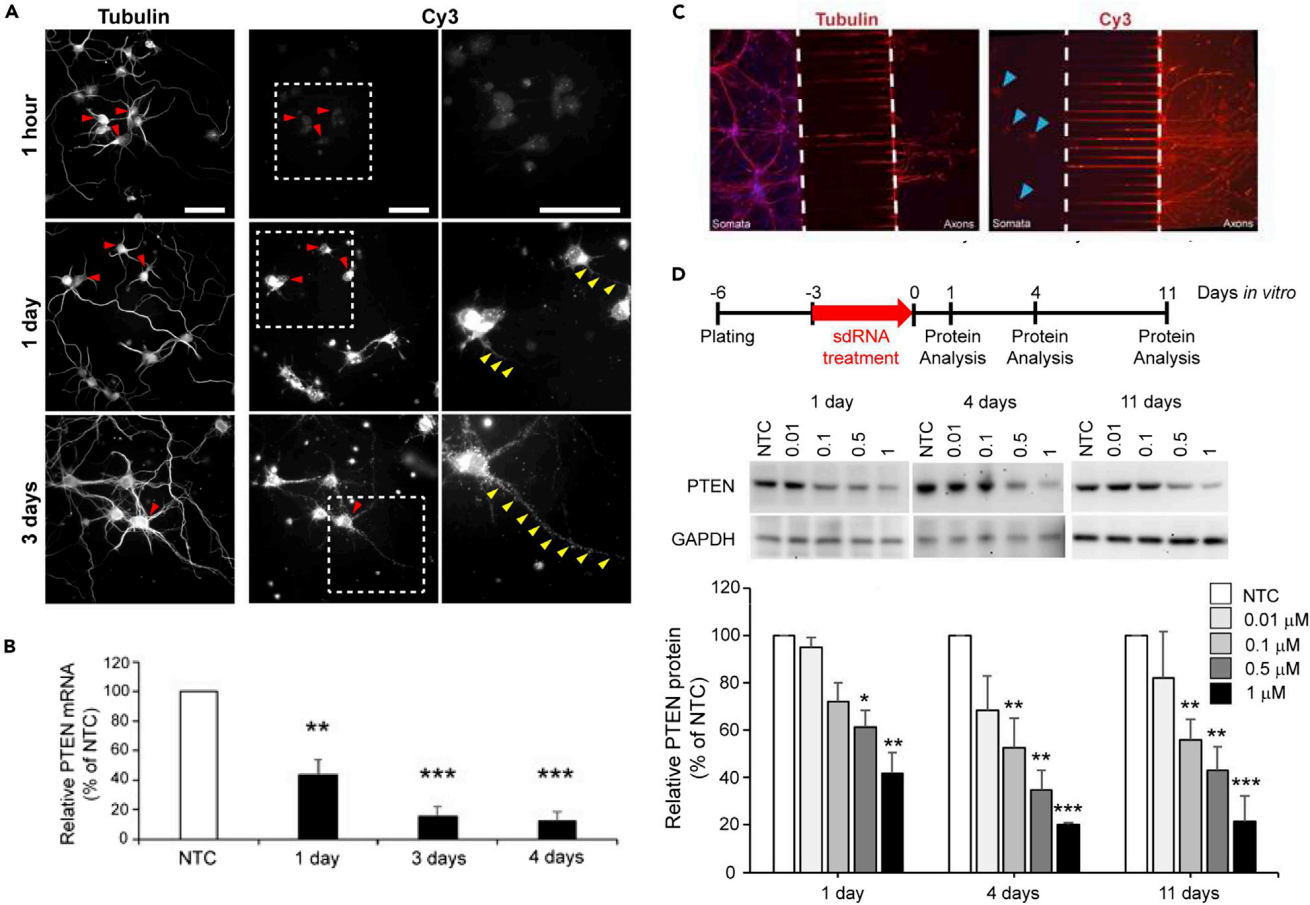
BA-434 inhibits PTEN protein expression in primary neurons (A) Uptake of sdRNA by rat primary neurons treated with BA-434-Cy3 and visualized at 1 h, 1 day, and 3 days. Left panel: immunostaining with β3 tubulin to show all neurites. Right panel: the same field showing Cy3-positive cells with the boxed region shown at higher magnification in the panel on the far right. Red arrowheads point to double-labeled cell bodies, yellow arrowheads highlight the neurites. Scale bar = 100 μm. (B) PTEN mRNA expression 1 day (n = 2), 3 days (n = 3) and 4 days (n = 4) after the administration of BA-434 (1 μM) to rat cortical neurons compared to the NTC control (F(3, 10) = 75.68, p < 0.0001; ∗ indicate *p*’s < 0.05 by Sidak multiple comparisons test). Shown are means of triplicate samples ±SEM from independent experiments. (C) Hippocampal neurons, plated in custom microfluidic chambers where axons are isolated from cell soma, show positive Cy3 fluorescence in the cell soma (arrowheads), 24 h after the application of BA434-Cy3 to the axon compartment. Left panel shows staining of all processes with β3 tubulin. (D) The top panel shows the treatment protocol to investigate PTEN expression at 1 day, 4 and 11 days after washing out BA-434 treatment. The middle panel shows representative dose-response Western blots at the three time points. The bottom panel shows the quantitation of the dose-response at 1 day, 4 days, and 11 days after washout. Plotted are means +SEM of three experiments. two-way ANOVA time: F(2, 30) = 5.46 p = 0.0095; dose: F(4, 30) = 27.88 p < 0.0001; ∗ indicate p < 0.05 by Dunnett’s multiple comparison test to NTC within each time.

Continuously cultured cell lines do not necessarily pose the same difficulties to experimental manipulations as do primary neurons, so we tested the ability of BA-434 to silence PTEN expression *in vitro* in cultures of primary rat cortical neurons. We first confirmed the uptake of sdRNA into cultured primary neurons using a fluorescent reporter generated by conjugating the red-fluorophore, Cy3, to the BA-434 guide strand (BA-434-Cy3) and determining via fluorescence microscopy that the transduction efficiency is quite high for all cell types in the culture (see Figure S2). Cells that have been plated in culture for 3 days have already established long neurites, and we examined the uptake of BA-434-Cy3 after exposing primary neurons for 1 h, 1 day, or 3 days (Figure 2A). By 1 day following treatment, fluorescence was readily observed and PTEN mRNA levels at this timepoint were already decreased substantially, as measured by qPCR (Figure 2B, p < 0.05 by ANOVA). The fluorescent signal continued to increase over 3 days (Figure 2A, bottom row) and ultimately became distributed to the neurite compartment. After 4 days of exposure, PTEN mRNA expression had been reduced by 94%.

A comparison of the Cy3 signal to βIII tubulin immunostaining of neurites showed that the neurites progressively accumulated punctate Cy3 fluorescence over time, but it was not clear if this was simply a result of neuritic uptake from the culture media or represented transport from the cell soma. To determine if sdRNA could be axonally retrogradely transported, we plated E17 hippocampal neurons in microfluidic chambers where the cell soma was spatially isolated from their axon tips and fluidically isolated by a hydrodynamic gradient to prevent bulk fluid flow from the axonal toward the somatic compartment (see Figure S3). The addition of BA-434-Cy3 to the fluidically isolated axon terminal compartment ultimately resulted in the appearance of Cy3 fluorescence in the neuronal soma within 24 h (Figure 2C), supporting the contention that these molecules can be taken up by the distal axon and subcellularly assorted via axonal transport.

In order to determine if PTEN knockdown was sustained after BA-434 is removed from the culture media, we performed a wash out experiment after treating the cells with different concentrations of BA-434 for 3 days. Western blots were performed on cellular homogenates prepared at 1, 4, and 11 days after removing BA-434 from the culture media. Quantitation of the PTEN protein showed a dose-dependent reduction (Figure 2D; p < 0.05 by ANOVA). There was a sustained knockdown of PTEN for 11 days with doses of 0.1, 0.5, and 1 μM.

### BA-434 tested *in vivo* reduces phosphatase and tensin homolog expression

**Figure 3:**
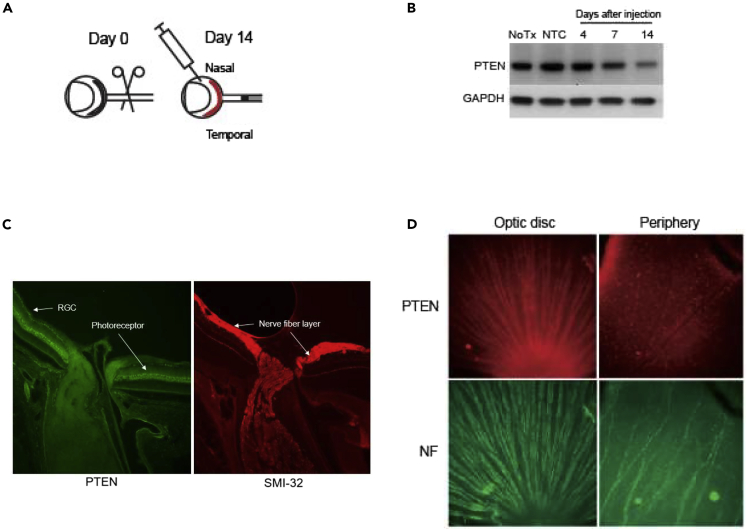
PTEN protein expression and RGC regeneration tested before the optimization of the sdRNA (A) Schematic representation of the injury and delivery paradigm to study RGC axon regeneration after adult rat optic nerve injury. (B) Western blot in pooled retinal extracts shows a reduction in PTEN protein out to 14 days after the intravitreal injection of 60 μg of sdRNA per eye. GAPDH was used as a control for loading. (C) PTEN and neurofilament staining of a central radial section of a control adult rat retina containing the optic nerve head demonstrates PTEN expression in both RGCs and in photoreceptors. (D) PTEN and neurofilament staining of retinal whole-mounts. PTEN is clearly detected in both RGC soma and axons close to the optic disc. Axonal PTEN staining is not evident at the periphery of the retina while RGC soma staining is still apparent.

Prior to proceeding to secondary screening, we verified the potential efficacy of these molecules *in vivo*. Induction of regeneration of retinal ganglion cell (RGC) axons after PTEN deletion has been extensively investigated (Kurimoto et al., 2010; Lim et al., 2016; Park et al., 2008), and therefore, we chose a rat optic nerve crush model (Figure 3A) to investigate the ability of these PTEN sdRNA compounds to stimulate axonal regeneration in the CNS. Biochemical analysis of pooled whole retinal protein extracts showed that a single, intravitreally delivered BA-434 dose reduces PTEN expression substantially out to 14 days post-injection (Figure 3B). Radial sections of a non-injected retina show that PTEN expression is largely limited to the retinal ganglion cells (RGCs) and photoreceptors (Figure 3C). Whole-mount staining of adult retina (Figure 3D) shows that PTEN expression is quite evident in both RGC somatic and axonal compartments near the optic disc, but as one moves to the peripheral retina the expression is largely only detectable in RGC cell bodies with neurofilament staining able to identify the axonal compartments both centrally and peripherally. It is important to note, mice are quite frequently used in preclinical studies of RGC regeneration, but mice and rats have a single nucleotide non-homology in the target sequence of our sdRNA. We tested the specificity of BA-434 to knock-down PTEN expression in mouse N1E-115 neuroblastoma cells and found that BA-434 had no impact on mouse PTEN expression, showing the critical selectivity of the sequence. Moreover, rat optic nerve is much larger than mouse optic nerve and the extent of regeneration observed in mice has never been achieved for rat, so it is a more robust model for clinical translation.

**Figure 4:**
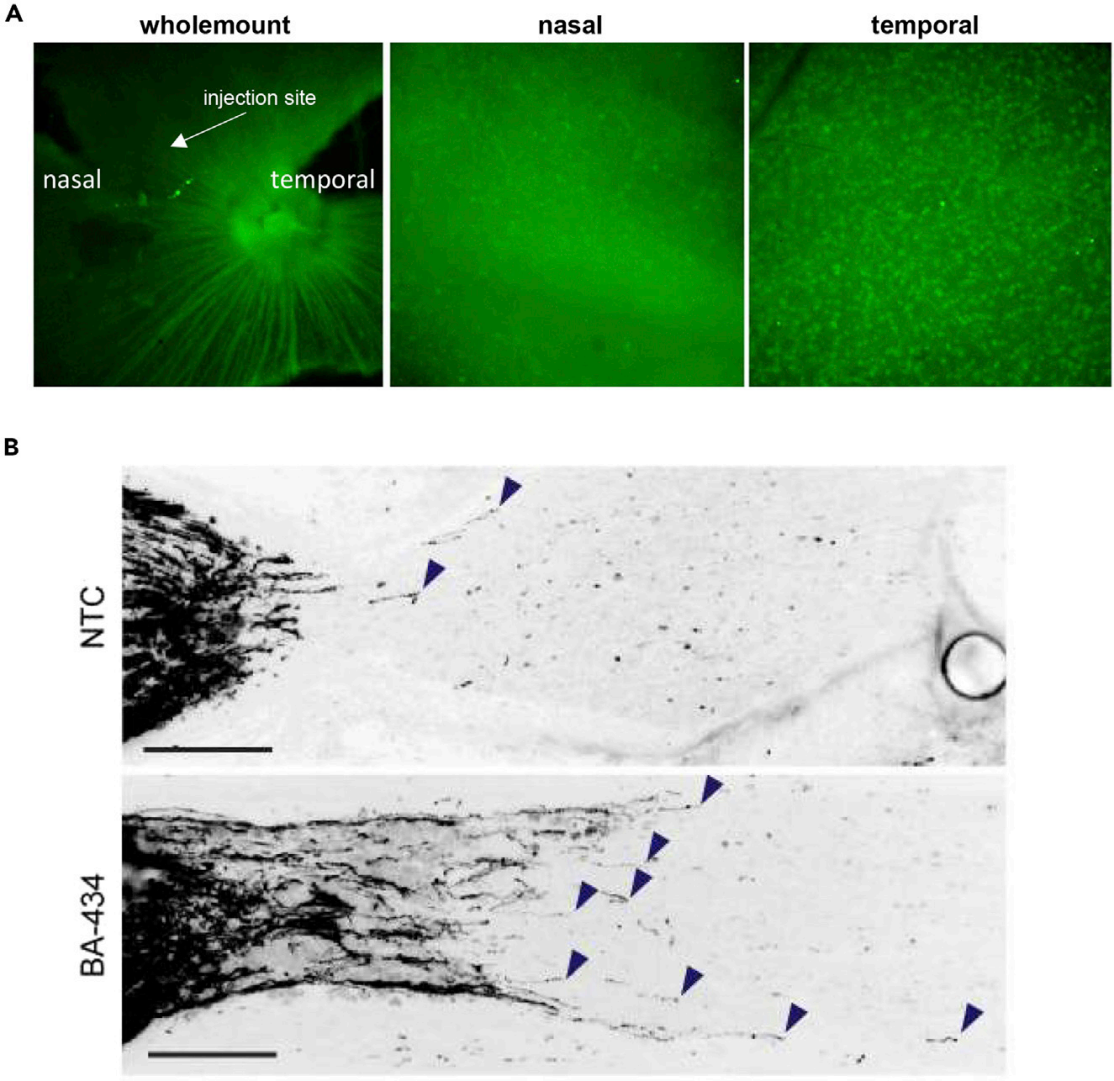
PTEN protein expression and RGC regeneration tested before the optimization of the sdRNA (A) Staining for PTEN expression in retinal whole-mounts 4 days after the injection of BA-434 shows a clear reduction in PTEN levels on the nasal side of the optic disc, correlating with the injection site. Images at slightly higher magnification taken at the retinal periphery show the reduction of PTEN expression in RGC soma on the nasal side (the injected side) versus the temporal side. (B) CTB labeled regenerating axons 14 days after either NTC treatment (top) or BA-434 treatment (bottom). CTB accumulates immediately proximal to the injury site. Bar = 100 μm.

Next, we characterized the expression pattern of PTEN in retinal whole-mounts 4 days following the injection of 30–60 μg of BA-434 into the vitreous chamber, with the choice of dose based on sdRNA dose-response experiments performed in mouse eyes (Byrne et al., 2013). Injection into the nasal aspect of the vitreous chamber leads to a marked reduction of PTEN fluorescent staining as detected by whole-mount preparation (Figure 4A), specifically in the area of injection, as imaged near the optic nerve head. Examination of peripheral retina regions shows that the RGC cell bodies of the nasal retina have greatly reduced PTEN expression as compared to the temporal retina, the side opposite to the injection of the sdRNA. Extraction of pooled retinal tissue at 4, 7, and 14 days after the injection and examination of signaling pathways downstream of PTEN by Western blotting also shows that sdRNA-driven reductions in PTEN impact its well-known downstream kinases and targets (see Figure S4). Identifying significant PTEN reduction in RGC soma, we next examined the effects this had on the regeneration of RGC axons in the optic nerve after crush injury. When the optic nerve was crushed approximately 1 mm posterior to the orbit and BA-434 injected into the vitreous, axon regeneration past the crush was observed at 14 days after injury/injection (n = 4), while in the non-targeting control (n = 4) few or no axons extended past the crush site (Figure 4B). These pilot regeneration experiments provided sufficient evidence of efficacy to proceed to secondary screening to create a pro-regenerative drug candidate based on this sdRNA technology.

### Secondary screening to optimize modified nucleotides

**Figure 5:**
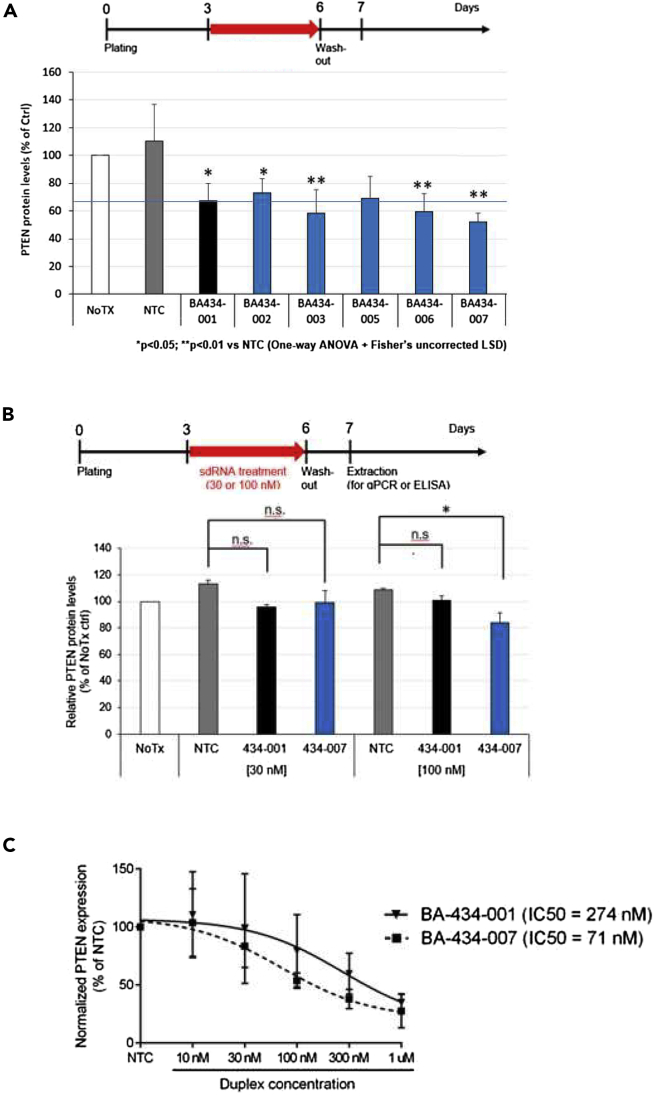
Screening for an optimized PTEN sdRNA (A) PTEN protein levels were measured using an ELISA on extracts prepared from primary cortical neurons treated according to the schematic shown. Blue line shows the efficacy of BA-434-001. Reductions compared to NTC were significant in all cases, with BA-434-007 appearing to be most effective. (B) Low dose testing shows that BA-434-007 is more effective than BA-434-001. Decrease in PTEN expression in primary rat cortical neuron cultures was measured by ELISA after a 4 days of treatment with either 30 or 100 nM of sdRNA. ∗p < 0.05 vs NTC by One-way ANOVA+ Fisher’s uncorrected LSD test. n.s. - not significant. (C) Dose-response for the reduction in expression of PTEN mRNA as measured by Q-PCR after treatment with either BA-434-001 or BA-434-007. Dose-response experiments were repeated three times and the IC50 was determined in each case. Comparison of the mean IC50 calculated (shown) from all three repeats was then compared statistically. Although BA-434-007 appears to be more effective at reducing PTEN mRNA, the IC50 data did not reach significance after the three repeats.

The development of a nucleic acid drug candidate requires appropriate properties to enhance stability and resist degradation. Adding 2′-*O*-methyl-, phosphorothioate, and 2′-fluoro- modifications enhance oligonucleotide stability (Byrne et al., 2013; Khvorova and Watts, 2017). For the primary screen, BA-434 contained modifications previously described for other sdRNAs (Ly et al., 2017). In the secondary screening process, we retained the BA-434 nucleotide sequence and changed the modifications made to the sense strand or antisense strand nucleotides (Table 1). We first screened the different constructs for PTEN knockdown in primary neurons (Figure 5A), and then took the best candidates and tested further duplex combinations (see Table S3). The best six from the first screen, and five new combined duplexes were then tested for their stability in different biological matrices as assessed by their ability to cause PTEN knockdown after initial incubation for 4 days at 37°C in rat plasma (see Figure S5A) or rat CSF (see Figure S5B). Following incubation in these different matrices, the samples were then added to neuronal cultures for 3 days before washing the cultures, changing the medium, and extracting them after an additional 24 h. From these tests, the top two candidates were the original BA-434, now called BA-434-001, and BA-434-007 (Table 1). These two candidates were then compared at very low doses (30 and 100 nM) for their efficacy in reducing PTEN expression in neuronal cultures following 4 days of treatment (Figure 5B). BA-434-007 produced a significant reduction in PTEN expression at 100 nM as compared to NTC treatment, while BA-434-001 did not. Dose-response curves for PTEN mRNA reduction in cortical neurons after 4 days of treatment were assessed by qPCR and the IC50s calculated for three independent experiments. Although the average IC50s (Figure 5C) do appear to show an increased efficacy for BA434-007 in reducing PTEN expression as compared to BA-434-001, the assay did not achieve significance.

**Table 1:** Secondary screening, first round of sdRNA modifications

ID	Strand	Sequence & modification pattern	Changes from BA-434	#F
**Sequence from primary screen**				

BA-434 001	Sense	5′mU.mA.G.mC.mU.A.mC.mC.mU.G.mU.mU.mA∗mA∗mA.TEG-Chl	Sequence BA-434 with modifications of primary screen	0
	Antisense	5′P.mU.fU.fU.A.A.fC.A.G.mG.fU.A.G.fC.fU∗A∗fU∗A∗A∗fU∗A		8

**Modifications to sense strand**				

BA-434 002	Sense	5’mU.mA.G.mC.mU.A.mC.mC.mU.G.mU.mU.**A**∗mA∗mA.TEG-Chl	2′O-Methyl removed	0
BA-434 003	Sense	5′mU.mA.**mG**.mC.mU.**mA**.mC.mC.mU.**mG**.mU.mU.mA∗mA∗mA.TEG-Chl	2′O-Methyl added to all nucleotides	0

**Modifications to antisense strand**				

BA-434 004	Antisense	5′P.mU.fU.fU.A.A.fC.A.G.mG.fU.A.G.fC.fU∗**mA**∗fU∗**mA**∗**mA**∗fU∗A	Fully modified by adding 3′ 2′O-Methyl groups	8
BA-434 005	Antisense	5′P.mU.fU.fU.A.A.fC.A.G.mG.fU.A.G.fC.fU∗**mA**∗fU∗A∗**mA**∗fU∗A	Fully modified except for position 17	8
BA-434 006	Antisense	5′P.mU.fU.**mU**.A.A.fC.A.G.mG.fU.A.G.fC.fU∗A∗fU∗A∗A∗**mU**∗A	Reduced 2′F modifications	6
BA-434 007	Antisense	5′P.mU.fU.**mU**.A.**m**A.fC.A.G.mG.fU.A.G.**mC**.fU∗A∗fU∗**m**A∗A∗**mU**∗A	Reduced 2′F modifications and increased stabilization	5
BA-434 008	Antisense	5′P.mU.fU.**mU**.A.**m**A.fC.A.G.mG.fU.A.G.**m**C.fU∗**m**A∗fU∗**m**A∗**m**A∗**mU**∗A	Fully modified while reducing 2′F modifications	5
BA-434 009	Antisense	**5′VP**.mU.fU.fU.A.A.fC.A.G.mG.fU.A.G.fC.fU∗A∗fU∗A∗A∗fU∗A	5′Vinyl-phosphonate replaced phosphate group	8
BA-434 010	Antisense	5′P.mU**∗**fU**∗**fU.A.A.fC.A.G.mG.fU.A.G.fC.fU**.**A∗fU∗A∗A∗fU∗A	Additional phosphorothioate modifications at the 5′ end	8
BA-434 011	Antisense	5′P.mU.fU.fU.**mA**.**m**A.fC.**mA**.**mG**.mG.fU.**fA**.**fG**.fC.fU∗**mA**∗fU∗**mA**∗**mA**∗fU∗**mA**	Fully modified with 2′O-Methyl and 2′F modifications	10

**Non-targeting control (NTC)**				

NTC	Sense	5′mU.mG.A.mC.mA.mA.mA.mU.A.mC.mG.mA.mU∗mU∗mU.TEG-Chl	Length and modifications matched to BA434-07	0
	Antisense	5′P.mU.fA.A.mU.fC.G.mU.A.fU.mU.fU.G.mU.fC∗mA∗A∗mU∗mC∗A∗G		5

Starting with the pattern of the parent duplex (BA-434-001) nucleotide modifications were added/changed (**bold**) or modifications were removed (**bold**) at specific positions in the sense and antisense strands. The general strategy was to increase stability and potency by introducing additional 2′-*O*-Methyl modifications (m), phosphorothioate modifications (∗), or vinyl phosphonate at the antisense 5′ end (VP) instead of a phosphate group (P) while at the same time reducing 2′-fluoro (F) modifications. #F heading = number of F modifications.

In order to more fully test the ability of these sdRNAs to advance as potentially useful therapeutics, we next assessed the impact of BA-434-001 and BA-434-007 on cell viability and the induction of immune activity. Primary human peripheral blood mononuclear cells (PBMCs) were treated with sdRNAs for 24 h before evaluating their impact on cell viability using an Alamar blue assay (see Figure S5C). Additionally, the induction of interferon-induced proteins with tetratricopeptide repeats (IFIT) (see Figure S5D), and the production of TNF in response to exposure to the sdRNAs were assessed (see Figure S5E). The toll-like receptor seven agonist Loxoribine served as a positive control for the induction of immune response-related transcripts. None of the sdRNAs negatively impacted the viability of PBMCs, and neither BA-434-001 nor BA-434-007 induced expression of IFIT or TNF. These data indicate that BA-434-007 has an acceptable profile for *in vivo* testing. Taking into account the *in vitro* data on potency (Figure 5), BA-434-007 was chosen as the best candidate to take forward.

### BA-434-007 sdRNA promotes long-distance axon regeneration

**Figure 6:**
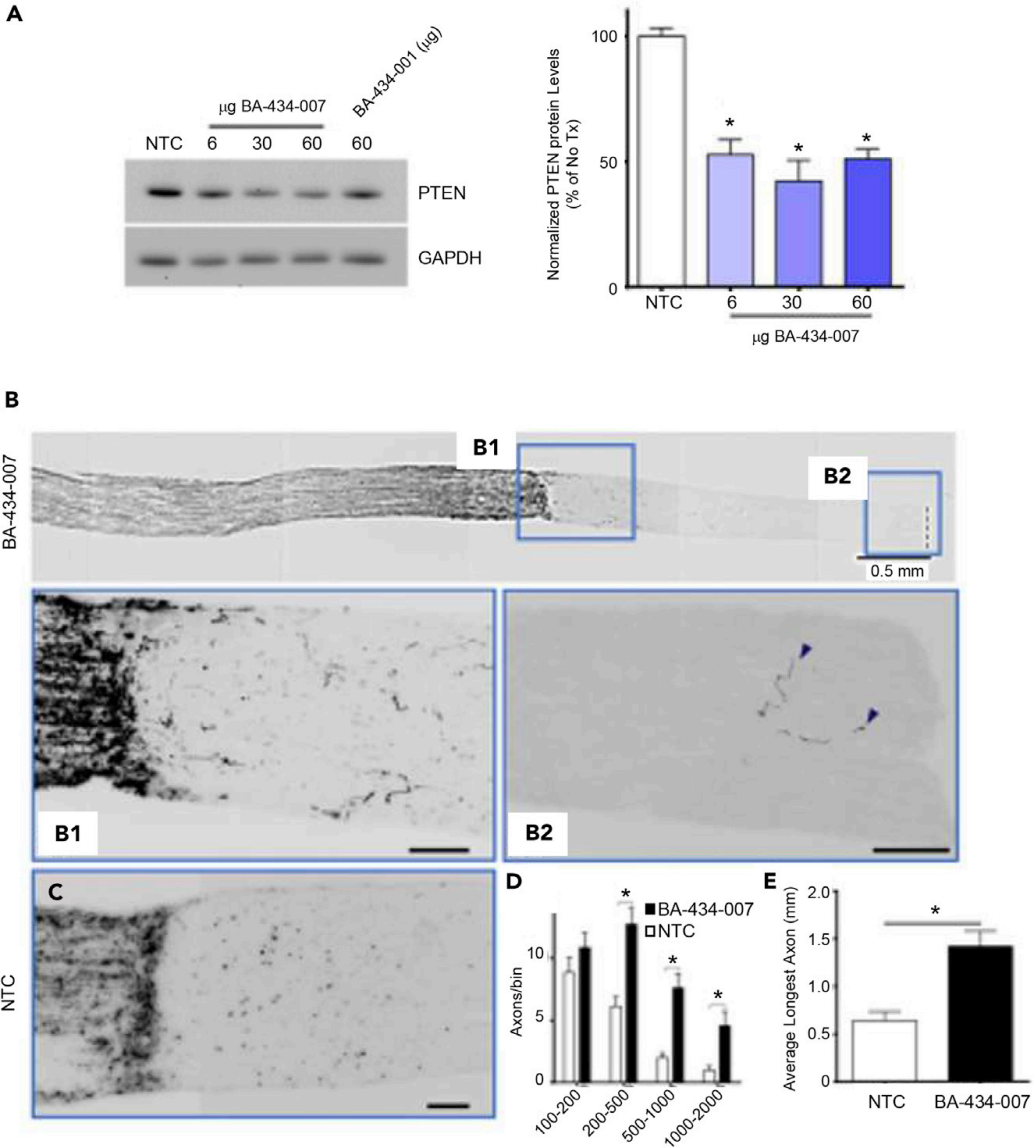
Efficient reduction of PTEN after intravitreal injection in adult rats (A) PTEN levels after the intravitreal injection of 6, 30, or 60 μg of BA-434-007 or 60 μg of NTC or BA-434-001. Samples from individual animals (n = 4/group) were run on Western blots and PTEN normalized to GAPDH. (p < 0.05 Dunnett’s multiple comparisons test). (B) Low magnification view of an optic nerve crushed 4 mm from the optic nerve head and treated with BA-434-007. B1 and B2 show higher magnification view of the boxed regions. Bar = 100 μM. (C) For comparison, an animal injected with NTC is shown at the same magnification as B1. (D) The number of regenerating axons counted in bins corresponding to distances of 100–200 μm, 200–500 μm, 500–1000 μm, and 1000–2000 μm past the crush. (Significance was determined by Sidak multiple comparison p < 0.05). (E) The longest regenerating axon was measured in three to five sections per animal. Treatment with BA-434-007 significantly increased regenerating axon length relative to NTC. (two-way ANOVA drug dose: F(1, 172) = 41.95, p < 0.0001; bin distance: F(3, 172) = 25.72, p < 0.0001; Sidak multiple comparison p < 0.05).

The two leading candidate sdRNAs were then directly compared for *in vivo* efficacy in reducing PTEN expression following intravitreal injection. Each of the three doses of BA-434-007 tested was effective in reducing PTEN protein levels compared to NTC (Figure 6A; p < 0.05 Dunnett’s multiple comparisons test). For comparison with BA-434-001, individual samples were pooled and run on a separate blot. We found that 30 and 60 μg of BA-434-007 produced a greater reduction in retinal PTEN protein expression than did 60 μg of BA-434-001.

To test the ability of BA-434-007 to promote axon regeneration, we subjected adult rats to optic nerve crush injury approximately 4 mm distal to the eye. A single intravitreal injection of the naked sdRNA compound was performed at the conclusion of the nerve injury surgery. Stimulation of axon regeneration in the optic nerve after an injury far from the adult eye is a much more challenging paradigm than typically undertaken because regenerative responses decrease as the distance of the injury from the RGC soma increases (Doster et al., 1991; Villegas-Pérez et al., 1993). The eyes were monitored for clinical signs and all eyes appeared normal without obvious signs of inflammation. Two weeks following this single-dose treatment, RGC axons were anterogradely labeled via intravitreal injection of fluorescent cholera toxin B subunit (CTB) and the extent of RGC axon regeneration was examined in longitudinal sections of the optic nerves. Long-distance axon regeneration past the crush site was observed in BA-434-007-treated animals (n = 5; Figure 6B) compared with few to no fibers in control animals treated with NTC (n = 6; Figure 6C). Regenerating axons were observed at distances of up to 3 mm beyond the crush injury, which is 7 mm total from the eye, and these axons followed a relatively tortuous path (Figure 6B2). Quantitation of the labeled axons binned per unit distance out to >2 mm from the injury site (Figure 6D), showed that a single dose of BA-434-007 significantly increased the number of regenerating axons that were present at all distances from the eye already at just 14 days after injury/treatment. Treatment with BA-434-007 produced regenerating axons that were significantly longer than those from animals treated with the control NTC sdRNA (Figure 6E). Treatment with the sdRNA was an absolute requirement for us to see any measurable regeneration (see Figure S6). To our knowledge, 7 mm from the eye is the furthest distance that adult rat retinal ganglion cell axons have been shown to extend after optic nerve injury, except for experiments where peripheral nerve grafts were used to enhance regeneration (Keirstead et al., 1985; Vidal-Sanz et al., 1987). The entirety of the optic nerve from the globe to the chiasm in rats is approximately 10 mm (Matheson, 1970).

## Discussion

Here we report on the use of sdRNA technology to generate a candidate antisense therapeutic that can be used without any additional excipients or carriers to reduce target protein expression in the CNS. We chose to target the reduction in PTEN expression as a means to test this technology platform for its ability to deliver a therapeutic that can stimulate axonal regeneration after injury in the CNS. This siRNA-cholesterol compound is capable of efficient penetration of the naked, double-stranded, RNAi-inducing oligonucleotide into central neurons, and can, after a single injection, specifically reduce target protein expression, in this paradigm, for at least 14 days post-injection. Axons do not regenerate spontaneously after injury in the CNS, and we chose PTEN because its suppression by gene knockout has been identified as one of the most promising approaches to overcome intrinsic barriers to neural regeneration (Liu et al., 2010; Park et al., 2008; Sun et al., 2011).

The first approval of an RNAi drug marked a new era for targeting previously undruggable targets. The approved drug, Patisiran, to treat polyneuropathy associated with hereditary transthyretin-mediated amyloidosis, delivers the RNAi to the liver via lipid nanoparticles (Kuehn, 2018). Beyond targeting the liver, translation of RNAi to reduce protein expression in other tissues has posed delivery and formulation challenges. Conjugating RNAi to cholesterol allows rapid entry into cells (Ly et al., 2017), protein knockdown is sustained for weeks to months (Alterman et al., 2015; Byrne et al., 2013), and a sdRNA compound recently completed a Phase 2 clinical trial for the treatment of hypertrophic scarring (NTC02030275).

Leveraging PTEN as a target to promote axon regeneration in the CNS has been validated by others through conditional knock-out approaches (Park et al., 2008; Geoffroy et al., 2016), delivery of AAV-shRNA (Lewandowski and Steward, 2014; Yungher et al., 2015; Park et al., 2008), using PTEN antagonist peptides (Ohtake et al., 2014). We chose to use adult rats rather than mice because the length of the adult rat optic nerve to the chiasm is approximately twice as long as that in the mice. In mice, AAV-shRNA against PTEN is much less effective than germline PTEN KO (Yungher et al., 2015). Comparing histological techniques of the AAV-shPTEN mice studied in different laboratories, the shPTEN-induced regeneration of mouse RGC axons shown with regular cryostat sections (Yungher et al., 2015) is much less dramatic than when thick section confocal imaging was used (Park et al., 2008). Therefore, it is difficult to compare the extent of regeneration between labs when different sectioning and labeling techniques are used. We used thin sections but not confocal microscopy. Importantly, the injury we induced was much further from the optic nerve head (4 mm) than the mouse studies, and RGC axon regeneration is more challenging when injury is far from the eye (Villegas-Pérez et al., 1993). It will be interesting to test other sdRNA constructs that are effective in mice to better compare with the transgenic mouse studies. Unlike PTEN knockout transgenic mice, the technique of using sdRNA reported here could be conducted in multiple doses or in combination with other strategies.

Exercise, which is known to improve functional recovery after SCI, also has an effect on PTEN expression, suggesting that it regulates activity-dependent plasticity (Abbott et al., 2006). Experiments with combination treatments in the mouse optic nerve injury model show that damping the PTEN signaling pathway together with neuronal stimulation can produce sufficient RGC growth to restore vision (Lim et al., 2016), at least in mice, where the visual pathway is short compared with rats or humans, Studies in chronic SCI injury models have suggested that PTEN knockdown even 1 year after SCI can be effective (Du et al., 2015) and delayed treatments are more therapeutically relevant. As a negative regulator of mTOR, PTEN deletion activates the PI3K/mTOR pathway, and is involved in regulating protein synthesis and cell survival (Liu et al., 2010; Park et al., 2008). Most likely, the future path of regeneration to restore vision will be combination therapies, which have been shown to be effective to promote the longest RGC regeneration (Kurimoto et al., 2010; Sun et al., 2011).

To promote the regeneration of RGCs to restore vision after trauma as a therapeutic strategy, a permanent reduction in PTEN might adversely affect vision because it leads to the progressive death of photoreceptors by loss of adhesion between cells of the retinal pigment epithelium (Kim et al., 2008). After SCI, the permanent deletion of PTEN leads to cortical expansion (Gutilla et al., 2016). People with PTEN hamartoma, a syndrome with autosomal dominant transmission, are hemizygous for PTEN and have macrocephaly (Blumenthal and Dennis, 2008), and therefore it is not known whether permanent deletion would be safe. We showed that the use of sdRNA to reduce PTEN expression in the retina was sustained for a duration of about 2 weeks. Extensive time course analysis would need to be completed in several species prior to translation to humans. Others have reported sdRNA can be used to repress protein expression for up to 1 month after application to mouse brain (Alterman et al., 2015). Our *in vitro* experiments with primary neurons showed dose-dependent silencing by passive uptake of BA-434 from the culture medium. The concentrations required to reduce PTEN mRNA in our experiments were in the range expected for the development of a therapeutic compound (Byrne et al., 2013) and the *in vitro* wash-out experiments showed a sustained, but still transient reduction of PTEN for 10 days after removal of the sdRNA from the culture medium, establishing that sdRNA is stable for at least that long *in situ*. We also looked at the distribution of sdRNA after uptake and found that it distributes to different neuronal compartments, and is retrogradely transported, at least over short distances. This raises the possibility of flexibility in delivery of sdRNA to neuronal tracts or dendritic fields, and not just to the area of the cell soma. Ultimately, the use of sdRNA technology to transiently reduce PTEN would avoid the problem of permanent deletion of PTEN and would likely provide a sufficient period of time for PTEN reduction to foster long-distance regeneration.

Mechanisms that regulate axon regeneration are sensitive both to the type of axon and the distance of injury from the cell body (Doster et al., 1991; Fawcett and Verhaagen, 2018). In adult rat RGCs, axotomy greater than 3 mm from the optic nerve head does not elicit the expression of growth-associated protein 43 (GAP-43), a protein exquisitely correlated with axon regeneration (Doster et al., 1991). Most studies of regeneration of RGCs crush the optic nerve near its exit from the posterior aspect of the eye. With our optimized compound BA-434-007, we tested axon regeneration after an optic nerve crush 4 mm from the eye, leaving a long length of uninterrupted axons in the optic nerve. Even with this demanding injury paradigm (Villegas-Pérez et al., 1993), the intravitreal application of a single dose of the naked sdRNA compound BA-434-007 was capable of promoting the regeneration of RGC axons up to a further 3mm or more beyond the site of injury.

Such lengths of regeneration require successful slow axonal transport of cytoskeletal proteins from the RGC cell bodies to the tips of the growing axons. Because axotomy without treatment arrests slow axonal transport (McKerracher et al., 1990) the sdRNA technology should be amenable to the use of repeat doses in larger animals, if necessary. With intravitreal injections of drugs, both small and large molecules, now routine for the treatment of retinal disease, this would also be clinically feasible. An important next step in the translation pathway to restore vision after optic nerve trauma will be to combine reducing PTEN with neuronal activity, which has been used to restore vision in mice (Lim et al., 2016). Rats have much longer visual pathways, but even in this species, visual responses have been restored after cutting the optic nerve through the use of peripheral nerve grafts to direct RGC axons to the tectum (Keirstead et al., 1985). Together with our therapeutically applicable strategy to promote RGC regeneration in the optic nerve using sdRNA, the audacious goal of restoring some degree of vision in people through RGC regeneration may become a realistic goal.

The successful development of a potential therapeutic drug candidate not only requires effective target engagement, but also key properties such as adequate drug stability, manageable formulation for clinical use, and minimization of unwanted side effects. The specificity and potency of RNAi drugs is an intrinsic property of the nucleotide sequence, while the other important features are directly controlled by modifications to the oligoribonucleotide backbone. Chemical modification of synthetic oligoribonucleotides reduces susceptibility to nucleases, and by testing different combinations of 2′-*O*-Methyl, phosphorothioate, and 2′-fluoro modifications we obtained compounds with stability to 3 days of exposure to either plasma or CSF. Furthermore, RNAi can induce immune stimulation, and certain sequence motifs are determinants of siRNA immunogenicity, particularly U-rich strands (Goodchild et al., 2009). Importantly, it is known that there are not only siRNA-dependent but also delivery vehicle-dependent effects on immune induction (Meng and Lu, 2017; Olejniczak et al., 2016). Being able to forgo the need for a delivery vehicle or complex in formulation owing to the self-delivering nature of these compounds is thus an important step in potentially minimizing unwanted effects. We tested BA-434-007 for immune activation in a cell-based assay with human PBMCs and found little induction of either TNF or IFIT. We also examined the rat eyes and retinae for any sign of inflammation, an important confounder in axon regeneration experiments. With BA-434-007, controlled for both endotoxin levels and overall purity, none of the rats showed any evidence of inflammation.

The concern that PTEN deletion to promote axon regeneration in the CNS is not translatable is somewhat tempered by the finding that deletion is not lethal in humans although it increases susceptibility to form tumors. PTEN is considered a tumor suppressor, and some have cautioned that despite robust regeneration when it is deleted, it is not a translatable target (Petrova and Eva, 2018). Not all PTEN mutations associated with tumors are loss-of-function, and sequencing data indicate that PTEN can act both as an oncogene and a tumor suppressor (Costa et al., 2015). The demonstration that permanent PTEN deletion in adult rat motor cortex does not induce malignant transformation 1 year after treatment (Gutilla et al., 2016) suggests the promise of transient silencing of PTEN expression with sdRNA technology as a potentially viable approach to promote repair in the CNS. We have used direct injection techniques rather than systemic delivery because sdRNA does not cross the blood–brain barrier without temporary disruption techniques (Gutkin and Peer, 2018). For therapeutic use in the CNS, modifications, such as the use of exosomes, improve CNS penetration (Didiot et al., 2016). Importantly, studies on regeneration in the visual system are broadly applicable to the CNS, providing relevance for the translation of this sdRNA technology to treat other forms of CNS trauma including spinal cord injury (Park et al., 2008).

### Significance

A self-delivering RNAi, delivered by direct injection and without the need for either extraneous excipients or an expression vector, can be used to transiently suppress the expression of important targets in the CNS and aid regeneration. Targeting PTEN with sdRNA technology effectively knocks down PTEN expression for a sustained period of several weeks *in vivo* and knockdown is not permanent, which is an advantage when a permanent change in the target pathway is not desired. Self-delivering RNAi compounds have excellent therapeutic potential to target proteins when small molecule or antibody approaches have failed or are not feasible.

### Limitations of the study

Optic nerve injury that occurs more than one or 2 mm from the globe is an intrinsically more difficult system in which to see regeneration. Direct comparison of these rat experiments to studies in mice is also more difficult, especially where the injury is performed close to the exit of the optic nerve from the globe. These studies were performed with a single intravitreal dose of PTEN sdRNA that may also not be optimal for identifying maximal regenerative effects.

## STAR★Methods

### Key resource table

**Table:** 

REAGENT or RESOURCE	SOURCE	IDENTIFIER
**Antibodies**		

Anti-PTEN, monoclonal	Cell Signaling	Cat. No. 9559; RRID:AB_390810
Anti-GAPDH, monoclonal	Santa Cruz	Cat. No. SC-365062; RRID:AB_10847862
Anti-βIII tubulin, monoclonal	Promega	Cat. No. G7121; RRID:AB_430874
Anti-neurofilament, monoclonal	BioLegend	Cat. No. SMI-32R
Anti-rabbit IgG, HRP-linked	Cell Signaling	Cat. No. 7074; RRID:AB_2099233
Anti-mouse IgG, HRP-linked	Cell Signaling	Cat. No.7076; RRID:AB_330924
Anti-mouse IgG (H + L), Alexa Fluor 488	Thermo Fisher	Cat. No. A-11001; RRID:AB_2534069
Anti-Rabbit IgG (H + L), Alexa Fluor 488	Thermo Fisher	Cat. No. A-11008; RRID:AB_143165
Anti-mouse IgG (H + L), Alexa Fluor 594	Thermo Fisher	Cat. No. A-11005; RRID:AB_2534073

**Chemicals, peptides, and recombinant proteins**		

Cholera toxin subunit B- Alexa Fluor 594	Thermo Fisher	Cat. No. C34777

**Critical commercial assays**		

PathScan® Total PTEN Sandwich ELISA Kit	Cell Signaling	Cat. No. 7882

**Experimental models: Organisms/strains**		

CD Sprague Dawley Rats	Charles River	www.criver.com
CD Sprague Dawley Rats	Taconic	www.taconic.com

**Oligonucleotides**		

see Table 1		
see Table S1		
see Table S2		
see Table S3		

**Software and algorithms**		

Prism Software	GraphPad Software	www.graphpad.com

### Resource availability

#### Lead contact

Further information and requests for resources and reagents should be directed to and will be fulfilled by the Lead Contact, Lisa McKerracher (lmck@bioaxonebio.com).

#### Materials availability

sdRNAs to PTEN are available to purchase from Advirna LLC. Contact LM at BioAxone for other requests.

### Experimental model and subject details

#### Experimental animals

For the *in vivo* regeneration studies, adult (200 to 220 g; 8 to 12 week old) female Sprague Dawley rats (Charles River or Taconic) were used. They were group housed under standard vivarium conditions. Preclinical experiments were performed at Tufts University School of Medicine and at McGill University in compliance with the NIH Guide for the Care and Use of Laboratory Animals. For studies of RGC regeneration, the number of animals was based on the expectation that regeneration should be observed in all animals 2 weeks after application of a test compound (Bertrand et al., 2005). The non-targeting control and BA-434-007 were shipped to the surgical site in numbered, masked tubes for blinding. For analysis, the scientists analyzing results were blinded to the sample identities until completion of each study. Only animals with technical problems during the surgery or processing were excluded, and they were excluded prior to result analysis.

#### Primary cortical neuron cultures

Primary cultures of rat cortical neurons were prepared from dissected E18 rat cortices using the tissue and dissociation and culture reagents provided by BrainBits, LLC. The genders of the animals from which these cortices were derived was not disclosed by the vendor.

### Method details

#### Study design

The study was designed to investigate the functional ability of sdRNA molecules targeting the decrease of PTEN expression in translationally relevant preclinical models of neurotrauma. The objective was to screen sdRNA sequences directed again PTEN for their ability to knock down PTEN expression in cultured human cells and primary rat neurons, and to test these compounds in adult rats for their ability to knock down PTEN *in vivo* and stimulate axon regeneration. We performed primary screening to select the best sdRNA for PTEN knockdown, then performed secondary screening to optimize nucleotide modifications for nuclease resistance and to minimize immunological stimulation from the test compounds. Finally, the lead candidate was examined in an optic nerve injury model.

#### Synthesis of sdRNA and luciferase assay

Design and selection of PTEN sdRNA sequences common to rat and human PTEN were determined by Advirna LLC. For the primary screening, sdRNAs were synthesized as separate guide and passenger strands (TriLink Biotechnologies; San Diego, CA) and dissolved in sterile RNase-, DNase-free water for injection (Calbiochem, 4.86505) at 200 μM concentration. Duplexes were annealed by mixing equal volumes of the strand solutions, followed by heating to 95°C for 5 min and allowing to cool gradually to room temperature. The quality of duplex formation was tested by using native gel electrophoresis (see Figure S1). The sdRNA solutions were stored at −80ᵒC. Prior to use, the sdRNA stock solution was heated to 37ᵒC for 5 min, vortexed, and briefly spun down. For secondary screening, sdRNA was made by Phio Pharmaceuticals with a MerMade 12 Oligonucleotide synthesizer, purified and de-salted and verified endotoxin-free, as described in the supplementary methods. Luciferase reporter plasmid was constructed by inserting PTEN targeting region into psiCheck2 plasmid (Promega, Cat# C8021; Madison, WI) downstream of the Renilla luciferase sequence. MAP4K4 sdRNA sequence (Byrne et al., 2013) was used as a positive control. The luciferase assay was performed as described before (Shmushkovich et al., 2018). Renilla expression was expressed as a percent of untreated control cells transfected with the reporter.

#### Dual luciferase screening assay

Luciferase reporter plasmid described above was used and MAP4K4 sdRNA sequence was used as a positive control. For screening, HeLa cells were transfected with the cloned plasmid with Fugene HD (Promega, Cat# E2311) according to the manufacturer’s instructions. HeLa cells were seeded at 2.5 × 10^6^ cells/10 cm^2^ dish in EMEM (ATCC, Cat# 30–2003; Manassas, VA) medium without antibiotics and transfected 6 h later with the plasmid at 2.5:1 FuGENE:DNA ratio. Cells were incubated for 16–18 h, washed 3 times with PBS, trypsinized and seeded into 96-well plate with pre-diluted sdRNA compounds at final concentrations of 1 μM sdRNA/10,000 cells/100 μL EMEM with 3% FBS. Cells were treated with sdRNA for 48 h, lysed with Glo lysis buffer (Promega, Madison, WI) and assayed for Renilla and Firefly Luciferase expression. For the assay, 20 μL aliquots of each lysate were added into duplicate opaque 96-well plates and mixed with either Renilla assay buffer (Promega) or Firefly luciferase assay buffer (25 mM glycylglycine, 15 mM MgSO_4_, 4 mM EGTA, 1 mM DTT, 2 mM ATP, 15 mM K_2_PO_4_, pH 7.8- and 1-mM D-Luciferin). The substrates D-Luciferin (Promega) and h-Coelenterazine (NanoLight, Pinetop, AZ) were added immediately prior to use. Luminescence was measured using a SpectraMax i3 (Molecular Devices, Sunnyvale, CA) at 480 nm to detect Renilla expression and at 550 nm to detect Firefly expression. Renilla expression was then normalized to Firefly expression and normalized Renilla expression for all tested compounds were expressed as a percent of untreated control cells.

#### qPCR

Rat pheochromocytoma PC-12 cells (ATCC, CRL-1721) were grown on collagen-1 coated vessels in RPMI medium containing a mix of 5% fetal bovine serum (FBS; Gibco) and 10% horse serum (Gibco). Neuronal phenotype was induced by shifting cells to RPMI supplemented with 1% FBS and 100 ng/mL nerve growth factor 7S (NGF; Sigma; St. Louis, MO) for at least 72 hours. For sdRNA treatment, cells were plated on 96-well collagen-1 coated dishes (Corning; Corning, NY) at 30,000 cells per well in the “induction” medium. HeLa cells were plated on 96-well plates at 6,000 cells per well in EMEM supplemented with 3% FBS. Cells were treated in triplicates with sdRNA compounds at specified doses for 48 h. After treatment, total RNA was isolated from cells using PureLink Pro96 purification Kit (Ambion) and used as a template in a probe-based multiplex one-step RT-qPCR. To setup the RT-qPCR, Quanta qScript XLT enzyme solution (VWR) was mixed with Taqman probes (Thermo Fisher Scientific; Waltham, MA) specific for either human (Hs02621230_s1, FAM) or rat PTEN (Rn00477208_m1, FAM) and as reference gene human GAPDH (4326317E, VIC) or rat Actb (Rn00667869_m1, VIC), respectively. PTEN expression was normalized to the corresponding reference gene expression and plotted as a percentage of the normalized expression in NTC sdRNA-treated cells.

#### Cultured primary neurons

Primary cortical neurons were obtained from embryonic day 17 (E17) rat cortices according to manufacturer instructions (Brain Bits, Springfield, Illinois). Cortices were incubated with a 2 mg/mL papain (Brain bits) solution at 30°C for 15 minutes. Papain solution was replaced by Hibernate solution (Brain Bits) and digested cortices were dissociated using fire polished glass pipettes. Cell suspension was centrifuged for 5 min at 1000 rpm and the pellet was resuspended in neurobasal active medium (Brain Bits) supplemented with 1% fetal bovine serum (FBS, Gibco) and 1% Penicillin-Streptomycin (Gibco/Thermo Fisher Scientific). Cells were plated on 24-well tissue culture plates coated for 1 hour with 100 μg/mL Poly-D-Lysine (Sigma-Aldrich) at 37°C. 200,000 cells were plated in each well containing neurobasal active culture medium supplemented with 1% FBS and 1% Pen/Strep and cultured at 37°C. After 3 days, the media was exchanged to neurobasal active medium/1% FBS containing NTC sdRNA (1 μM) or different concentrations of BA-434 sdRNA (0.1, 1, 0.5 or 1 μM), respectively. After 3 days, medium was exchanged twice to washout sdRNAs using neurobasal active medium supplemented with 1%FBS. At 1, 4, 11 days after sdRNA removal, which is equivalent to 4, 7 and 14 days after sdRNA addition, primary cortical neurons were extracted from tissue culture wells using RIPA buffer (Boston Bioproducts, Ashland, MA) supplemented with complete protease/phosphatase inhibitor (HALT, Thermo Fisher Scientific) and lysed for 10 min at 4°C followed by centrifugation at 10,000 rpm for 10 min (4°C). After centrifugation, supernatants were mixed 3:1 with 4× Laemmli buffer (Biorad, Hercules, CA) and boiled for 5 min at 95°C. Denatured samples were electrophoresed under reducing conditions on 10% Bis-Tris-polyacrylamide gels (Novex/Thermo Fisher Scientific) in 3-(N-morpholino)propanesulfonic acid (MOPS) buffer (Novex/Thermo Fisher Scientific). Gels were transferred overnight (4°C) onto 0.25 μm PVDF membranes (EMD Millipore) using transfer buffer (Novex/Thermo Fisher Scientific) supplemented with 10% methanol and 0.1% antioxidant (NuPage). After protein transfer, the membranes were blocked with 5% BSA in TBS-Tween (0.1%) for 1 h at room temperature and then incubated with primary antibody against PTEN (1:2500; Cell Signaling, Cat# 9559) and glyceraldehyde 3-phosphate dehydrogenase (GAPDH, 1:5000; Santa Cruz, Cat# 365062) overnight at 4°C. After washing 3× with TBS-Tween (0.1%), the membranes were incubated with goat anti-mouse (1:10,000; Cell Signaling, Cat# 7076) or anti-rabbit (1:10000; Cell Signaling, Cat# 7074) secondary antibodies conjugated with horseradish peroxidase for 1 h at room temperature, respectively. Protein levels were revealed using chemiluminescence substrate (Super Signal West, Thermo Fisher Scientific) in conjunction with a FluoroChem SP Imaging System. The PTEN antibody was validated by the vendor and by determining specificity in tissue homogenates from cultured cells and rat tissue where a single band of the correct molecular weight was observed.

For studies of sdRNA uptake into cultured cells, neurons were cultured as described above. After 3 days, the media was exchanged to neurobasal active medium/1% FBS containing 300 nM Cy3-labelled BA-434. Cells were fixed with 4% PFA at 3 h, 24 h or 72 h after treatment exposure. If also stained with anti-beta-3 tubulin antibody, the procedure was as described below.

#### Microfluidic chambers

Custom microfluidic chambers were made using a custom designed photoresist surround template generated using soft photolithography (Phenomyx; Cambridge, MA). Microfluidic neuron growth chambers were cast from the custom templates using de-gassed Sylgard 184 Elastomer. After overnight curing, cell plating chambers were cut adjacent to either side of a series of parallel, 6 μm deep grooves. Chambers were washed, sterilized in alcohol, dried and then applied to sterile, rectangular microscope cover glasses that had been pre-coated with poly-d-lysine and laminin. Hydrostatic pressure differences can be used to restrict bulk fluid flow from one chamber towards the other by simply maintaining a lower volume of liquid in one chamber as compared to the other. Neurons were plated in one side of the device only and allowed to extend axons through the microgrooves towards the other chamber over the course of 7–10 days in culture with half volume of media changes every 48 to 72 hours, with equal media volumes being maintained during the axonal outgrowth phase of the experiment. For sdRNA transport studies, day 10 cultures were fed, with the soma compartment given a greater volume of media final to maintain hydrostatic pressure driving bulk fluid flow towards the axonal compartment. The sdRNA was added to the axonal compartment and the cultures maintained for an additional 24 hours before the cells were fixed with 4% paraformaldehyde and imaged by fluorescence microscopy.

#### Neurite outgrowth assay

PC-12 cells were plated on collagen-1-coated 10 cm dishes (70 μg/mL; Corning, Corning, NY) and grown until 90% confluency with RPMI medium, 10% horse serum, 5% FBS and 1% Pen/Strep. Cells were collected by trypsinization, then plated on poly-D-lysine-precoated 12 mm coverslips (Corning Biocoat), pre-coated with 5 μg/mL mouse laminin protein (Thermo Fisher Scientific), and placed in RPMI medium, 1% FBS, 1% Pen/Strep and 1 ng/mL NGF (Peprotech, Rocky Hill). After 3 days in culture, sdRNA was added to the culture medium at 1 μM and cells were further cultured for 3 days followed by a one-day washout period. Medium was aspirated from wells and a prewarmed 4% PFA solution was added to each well for 15 min. After fixation, cells were blocked for 1 h at room temperature with 4% BSA blocking solution followed by 1 h incubation with mouse anti-beta-3 tubulin antibody (1:1,000; Promega, Madison, WY). After thorough washing, coverslips were incubated for 30 min (room temperature) with goat anti-mouse secondary antibody conjugated to Alexa 488 (1:500; Thermo Fisher Scientific). Coverslips were mounted with Diamond Antifade supplemented with DAPI nuclear counterstain (Life Technologies) on microscope slides and dried overnight at room temperature. Ten independent areas per coverslip were imaged with a Nikon Eclipse E800 epifluorescence microscope using standard acquisition parameters. The number of cells with neurites and number of neurites per cell were counted on the photographs and quantified by a scientist blinded to the treatment conditions.

#### Primary sdRNA sequence screening *in vitro* and *in vivo*

Twenty sequences (see Table S1) were screened by luciferase reporter plasmid construct (supplemental methods) and 6 of 20 sdRNAs were selected for secondary screening (Table 1), with the BA-434 sequence selected as the optimal sequence. PTEN protein knockdown was studied by Western blot or ELISA assay in cell homogenates, and by Western blot in retinal homogenates. For Western blots, PTEN protein levels were normalized to expression of GAPDH as a control. The polyclonal antibodies to PTEN used in this study were validated by detecting loss of signal upon PTEN RNA knockdown, and by confirming expected molecular weight on Western blots.

#### Secondary sdRNA modification screening *in vitro* and *in vivo*

Individual nucleotides within the parent BA-434 sequence were modified (see Table S2) and PTEN knockdown was assessed in primary cultures. Further analysis included examination of sdRNA stability in plasma and CSF and induction of immunological response (See supplementary methods). Human peripheral blood mononuclear cells (PBMCs) were treated with 0.01, 0.1, or 1 μM sdRNA or positive controls Loxoribine (Lox) and Poly I:C (lipid-mediated transfection). Twenty-four hours post transfection IFIT and TNF expression was determined by the branched DNA assay (Affymetrix; Santa Clara, CA) according to manufacturer’s protocols. Data are displayed as fold IFIT or TNF expression over untreated cells. Error bars represent the standard deviation of 3 biological replicates.

#### Optic nerve injury and RGC regeneration

For optic nerve crush, female Sprague-Dawley rats (200–220 g) were obtained from Taconic (experiments at Tufts) or Charles River Laboratories (experiments at McGill). Female rats were chosen for screening to compare with previous published results (Bertrand et al., 2005; Ellezam et al., 2002; Lehmann et al., 1999) and to minimize the number of animals required. Rats were anesthetized with isoflurane, injected subcutaneously with buprenorphine, and an incision was made superior to the left orbit. Under a surgical microscope, the extra-ocular muscles were resected to expose the underlying optic nerve. The nerve was crushed approximately 1 mm behind the optic nerve head during primary screening or 4 mm behind the optic nerve head during secondary screening. It is more challenging to elicit axon regeneration with experimental treatments when the crush is further from the eye (Villegas-Pérez et al., 1993). The crush was with fine forceps (Dumont #5) for 10s, and care was taken to avoid damaging the ophthalmic artery. Vascular integrity of the retina was assessed by a fundus examination.

For intravitreal injections, a small puncture was made in the sclera with a 30-gauge needle and 5 μL of compound injected into the intravitreal space using a Hamilton syringe. The needle was held in place for ∼2 minutes to avoid reflux and the puncture was sealed with surgical glue. Intravitreal injections of cholera toxin subunit B conjugated with Alexa Fluor 594 (1 mg/mL, 5 μL, Thermo Fisher Scientific cat# C34777) were performed on day 13 following injury. Rats were transcardially perfused with buffered 4% paraformaldehyde (PFA) on day 14. Optic nerves were harvested on day 14 and post-fixed in 4% PFA for 2hr. Optic nerves were cryoprotected in 30% sucrose for 48 hours and embedded in OCT or M1 Matrix. Longitudinal sections (14 μm) were collected onto SuperFrost Plus slides (FisherBrand), washed with dH_2_O and imaged with a Nikon Eclipse E800 epifluorescence microscope.

In prescreening PTEN protein levels by Western blot, 3 animals were used per time point. For prescreening regeneration, optic nerves labeled with CTB were examined after treatment with BA-434 (n = 4), PBS (n = 3) or NTC (n = 4). In secondary screening for dose-response of PTEN protein reduction, rat eyes were injected with BA-434-007 (6 μg, 30 μg, or 60 μg in 5 μL; n = 4 rats per group), NTC (60 μg in 5 μL; n = 4), or no treatment (n = 4). Seven days after injection, retinas were collected, and flash frozen in liquid nitrogen. Protein was extracted from retinas and PTEN expression assessed by Western blot and comparison relative to NTC-injected animals. To assess RGC axon regeneration, 30 μg in 5 μL of BA-434-007 (n = 5) or matched NTC (n = 6) were used. The number of regenerating axons visualized with CTB were counted in 3-5 sections per rat in bins of 100–200 μM, 200–500 μM, 500–1000 μM, and 1000–2000 μM from the lesion site.

#### Immunostaining of whole mounts

Removal of the retina and preparation of whole mounts from the rat eye was performed as follows. The eye is removed from the perfused animal and is pinned down in a PBS filled dish, and a suture used to mark the dorsal surface. Excess connective and other extraocular tissues are removed from the eye and then, the cornea is removed. With the anterior tissue removed, the lens is carefully removed from the inside of the globe. A deep cut is made along the long dorsal axis of the globe to serve as a positional registration mark, and three other, shorter, evenly spaced cuts are made to mark the eye, and the retina, into 4 quadrants. These 4 quadrant cuts will also ultimately allow the retina to lay flat on a slide after processing. Using micro-Vannas scissors to get under the retina, the optic nerve is cut and the retina floats free from the eye cup. If a peripheral region is adhering to the sclera, that region is trimmed to allow the retina to be liberated from the eye tissue (Villegas-Pérez et al., 1993). Retinal whole mounts were processed for immunocytochemistry in 1.5 mL Eppendorf tubes with 100 uL of fluid. The retinas were rinsed in PBS with 2% Triton X-100 (TX), blocked in 4% BSA in PBS with 2% TX and then incubated overnight at room temperature with antibody in 4% BSA in PBS with 2% TX. After rinsing in PBS + TX and incubation in second antibody for 2 hours, the retinas were rinsed, wet mounted on slides with a drop of FluoromountG (Southern Biotech) and a coverslip sealed with nail polish prior to fluorescence microscopy. Primary antibodies used were rabbit anti-PTEN (1:100, Cell Signaling, Danvers, MA), or mouse anti-neurofilament (1:200, BioLegend; San Diego, CA). Secondary antibodies were goat anti-rabbit Alexa 488 and goat anti-mouse Alexa 594 secondary antibodies diluted 1:500.

#### Scaled up synthesis of BA-434-001, BA-434-007 and length-matched NTC

Therapure^TM^ ribo phosphoramidite monomers were procured from Thermo Fisher Scientific (Milwaukee, WI). All synthesis solid supports (CPG) with the first 3′ residue attached were obtained from LGC Biosearch Technologies (Petaluma, CA). Passenger strands containing sterol were synthesized from CPG functionalized with cholesterol-triethylene-glycol-glycerol succinate. Phosphitylation reagent for 5′-phosphorylation was procured from ChemGenes (Wilmington, MA). All other synthesis reagents and solvents were obtained from Millipore-Sigma (Burlington, MA). Other chemicals and solvents for downstream processing were purchased from Sigma Aldrich (St. Louis, MO) and were used without any purification or treatment. Oligoribonucleotides were synthesized using a MerMade 12 Oligonucleotide synthesizer (BioAutomation, Plano TX) using phosphoramidite chemistry. Synthesized oligonucleotides were cleaved from support and the protecting groups were removed by successive treatments of 3:1 aqueous ammonia:ethanol (40°C, 20h) and triethylaminetrihydrofluoride (DMSO, 60°C, 2h). Crude oligonucleotides were precipitated with isopropanol and centrifuged to obtain a pellet. Crude product was then purified on GE Akta Purifier UPC100 using ion-exchange chromatograph (Source 15Q, 20mM NaH_2_PO_4_, 15% CH_3_CN, 1M NaBr, Gradient 20–60%B over 30 column volumes) and fractions were analyzed by reverse-phase ion-pair chromatography on Shimadzu high-performance liquid chromatography (HPLC). Selected fractions were pooled and desalted by Tangential flow filtration using Sius PES 3000 MWCO (High Purity New England, Providence, RI) and evaporated to dryness on GeneVac Personal Evaporator (SP Industries, Warmingster, PA). The purity and MW were determined by HPLC analysis (Shimadzu Prominence; XBridge OST C18 column, 25mM hexylammonium acetate in water and 25mM hexylammonium acetate in acetonitrile gradient, 60°C) and ESI-MS analysis using Promass Deconvolution for Xcalibur (Novatia, Newtown, PA). All oligos had endotoxin levels <2 EU/mL by endosafe-PTS^TM^ assay (Charles River Laboratories International, Inc. Charleston, SC).

### Quantification and statistical analysis

Data were presented as mean ± S.E.M. unless otherwise noted. Prism V6.07 (GraphPad Software, San Diego, CA) was used for all statistical analyses. Unpaired student’s t-tests were used to analyze the number of PC-12 cells with neurites *in vitro* (Figure 1E) and longest regenerating axon after optic nerve injury (Figure 6E). A one-way ANOVA with Dunnett’s post-test was used to analyze the PTEN protein levels in rat cortical neurons after treatment with BA-434-007 (Figure 2D). A two-way ANOVA with Šidák post-test was used to analyze axon regeneration between treatments and at binned distances after optic nerve injury (Figure 6D). Results were considered statistically significant for p values less than 0.05.

## Data Availability

Data: Compiled data reported in this paper will be shared by the lead contact upon request. Code: This paper does not report original code. Any additional information required to reanalyze the data reported in this paper is available from the lead contact upon request.
