# Supplementary material for: Self-delivering RNAi compounds as therapeutic agents in the central nervous system to enhance axonal regeneration after injury
Source: iScience. 2022 May 10;25(6):104379. doi: 10.1016/j.isci.2022.104379 (PMC9127586; doi:10.1016/j.isci.2022.104379)
Supplement: Document S1. Figures S1–S6 and Tables S1–S3 [file mmc1.pdf]

iScience, Volume *25*

Supplemental information

Self-delivering RNAi compounds as therapeutic agents in the central nervous system to enhance axonal regeneration after injury

Sarah A. Woller, Joerg Ruschel, Barbara Morquette, James Cardia, Dinxue Yan, Katherine Holton, Taisia Shmushkovich, Emily Niederst, Karen Bulock, Alexey Wolfson, Matthew Abbinanti, Alyson E. Fournier, Lisa McKerracher, and Kenneth M. Rosen

Supplemental Figure 1

A

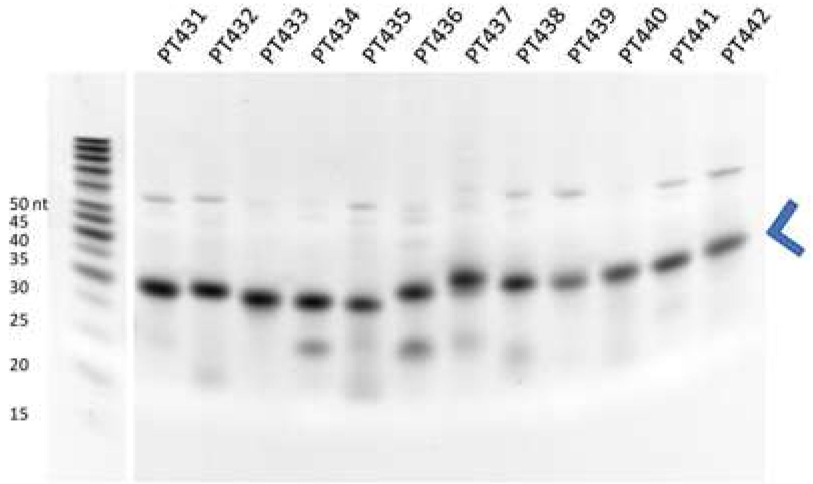

B

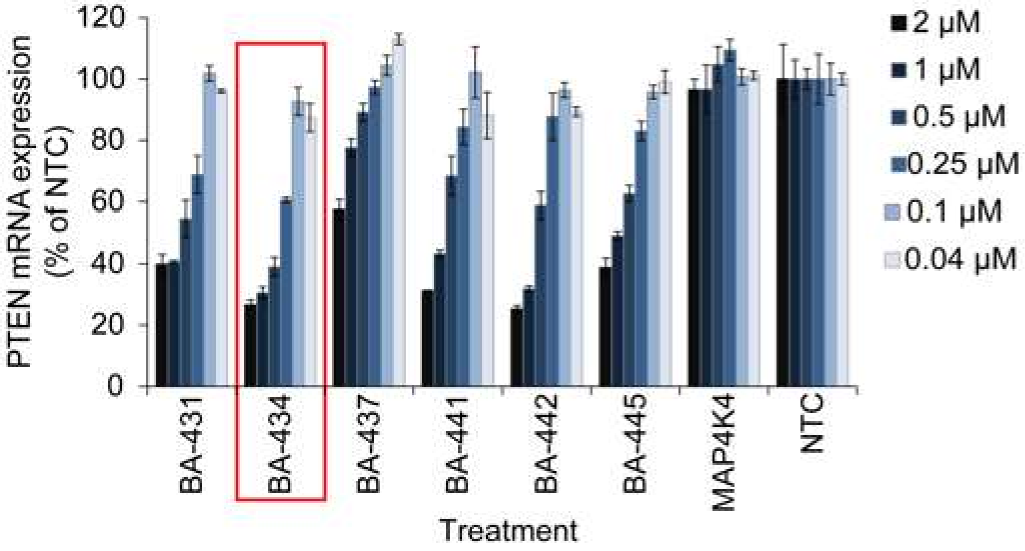

Figure S1. Structural and Functional Screening of Initial PTEN-targeting sdRNA. Related to Figure 1. A - Confirmation of RNA duplex formation. Prior to screening RNA and protein knockdown by self-delivering sdRNA, the quality of the duplex formation was verified. Duplex formation is verified in 20% native TBE gels, and the major bands represent the duplexes (arrowhead). The slight excess of single stranded RNA that is detected should not affect silencing. The 6 best sdRNAs from the primary screen were tested at concentrations of 0.1, 0.25, 0.5, 1 and 2 µM of test compound added to cultures of PC-12 cells treated for 48 hours, along with MAP4K4 and NTC sdRNAs as negative controls. PTEN expression was normalized to NTC and plotted as a percentage of NTC treated cells. All values are means ± S.E.M of triplicate measurements.

Supplemental Figure 2

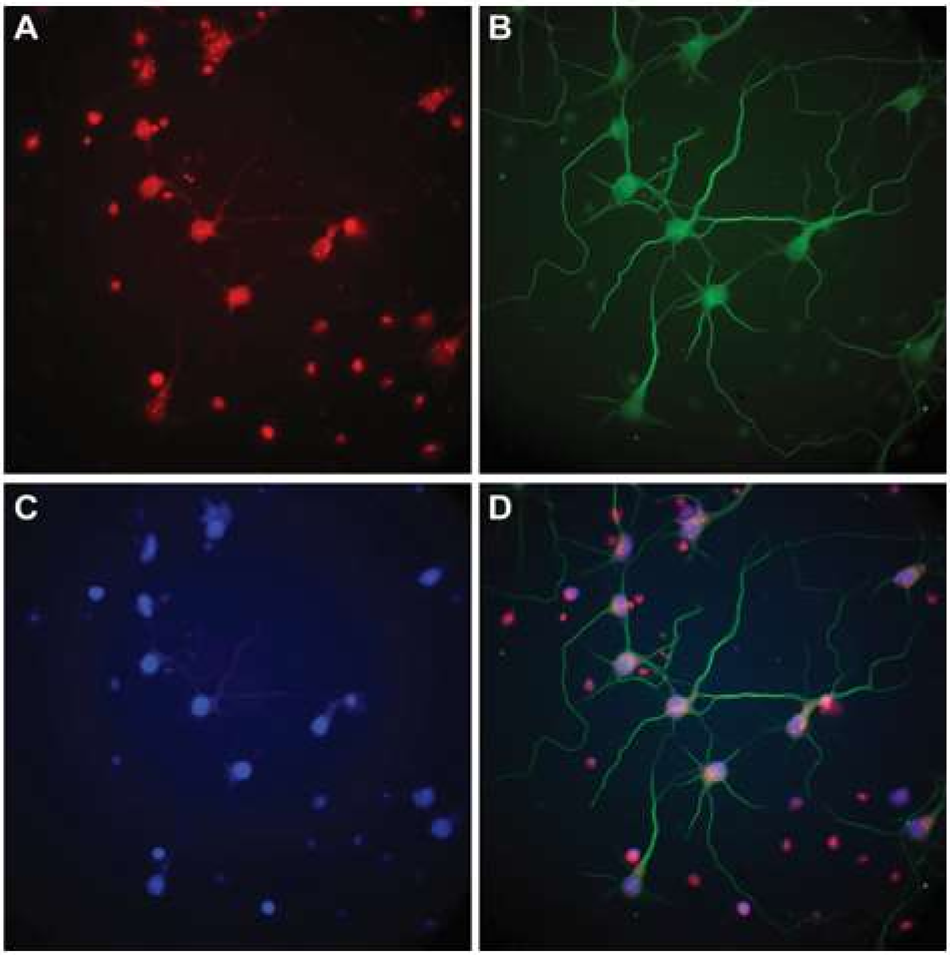

Figure S2. Uptake of sdRNA into cultured primary neurons. **Related to Figure 2.** Primary cortical neuron cultures were treated with (A) fluorescent conjugated BA434-Cy3 and then imaged for (B) anti-neurofilament and (C) Hoechst 33342 for DNA and all three images were merged (D). Transduction efficiency assessed in micrographs of the triple-stained cultures showed 100% transduction under our culture conditions with a dose chosen based on saturation conditions (Alterman et al., 2015). Our findings are agreement with published results with other sdRNA constructs (Alterman et al., 2015). .

Supplemental Figure 3

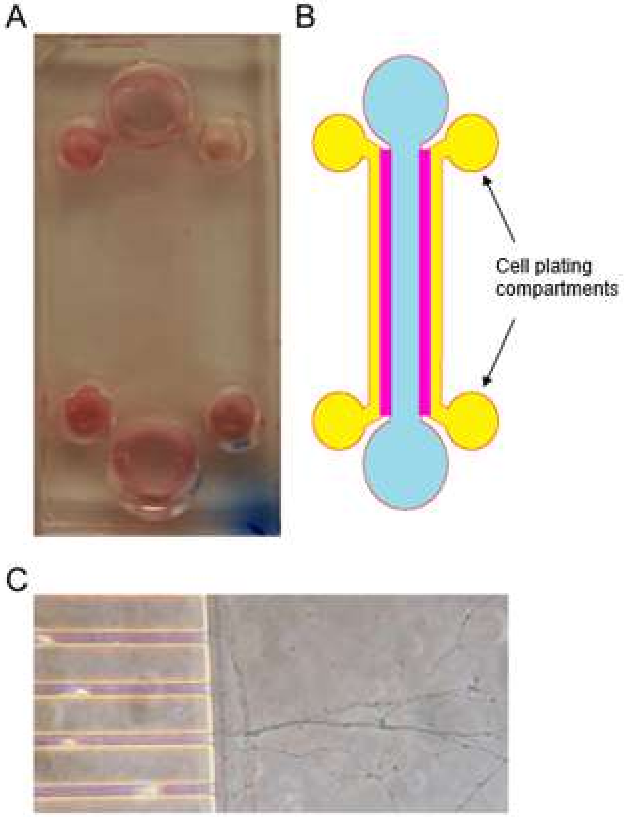

Figure S3. Construction of microfluidic chambers. Related to Figure 2.

(A). Microfluidic chambers were cast on a custom template generated by photolithography using Sylgard 184 (Vinyl terminated polydimethylsiloxane) to produce a device with a series of parallel 6 µM deep grooves between chambers. (B) Schematic figure showing wells and channels identifying cell soma compartments in yellow, axon compartments in cyan, and the capillary channels in magenta. (C) Phase micrograph of E17 rat hippocampal neurons that had been plated in the cell compartment and allowed to extend processes for 7 to 10 days into the axon compartment.

Supplemental Figure 4

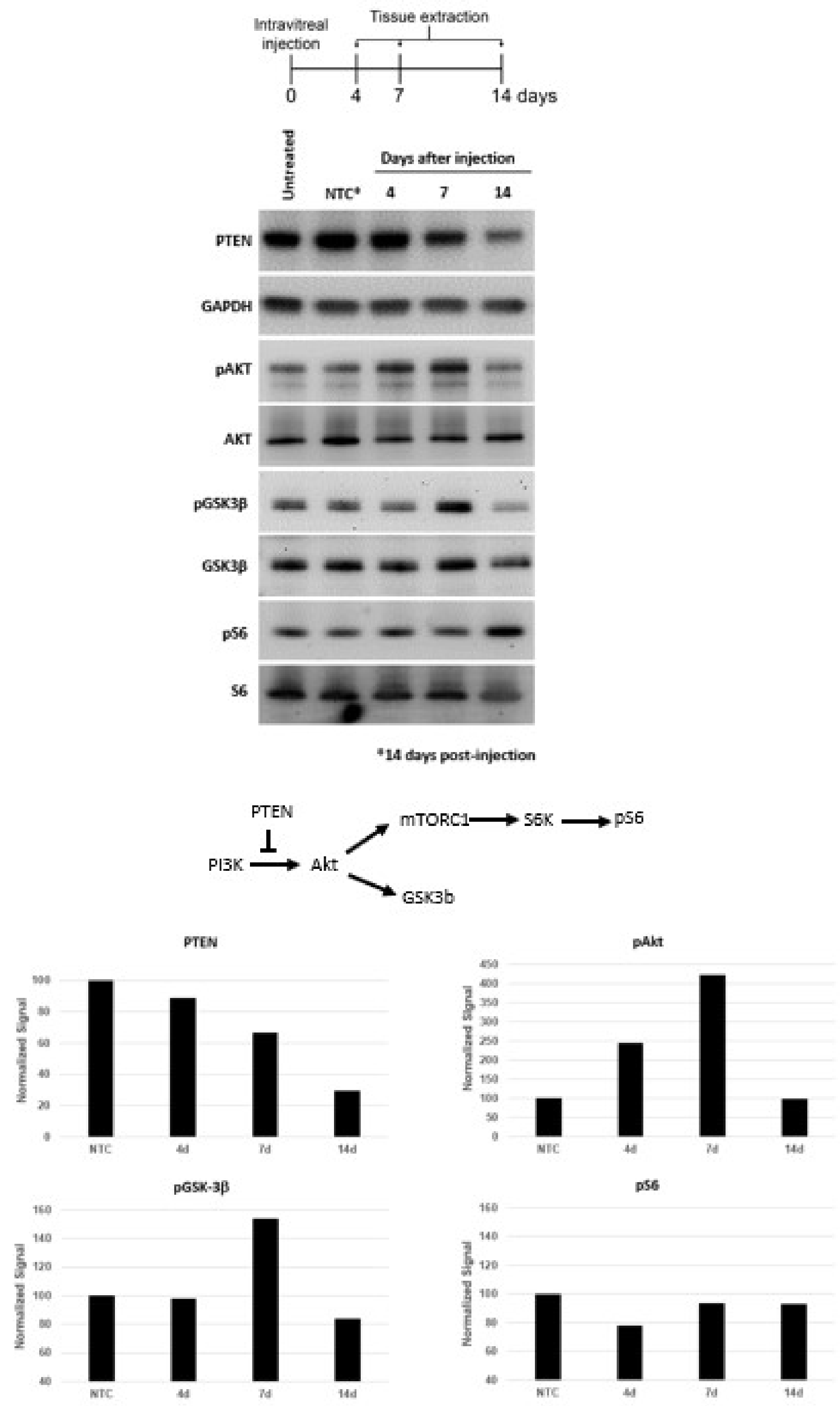

Figure S4. PTEN knockdown impacts its downstream signaling pathways. Related to Figure 4. Western blot analysis of PTEN and its related downstream intracellular signaling molecules after a single injection of BA434-007 in an adult rat. Pooled retinas (n=4) for each timepoint were analyzed for activation of Akt (increased pSer473 Akt) and its direct downstream target GSK-3β and the more distal S6 ribosomal protein. Akt activation appears to reach maximum at d7 and then begins to decline. Further downstream GSK-3β showed the same trend; a change in S6 was not observed. GAPDH shows equal protein loading on each lane. Companion blots for the total protein level for each of the 3 downstream target are shown. Densitometric analysis for each of the proteins is shown at the bottom.

Supplemental Figure 5

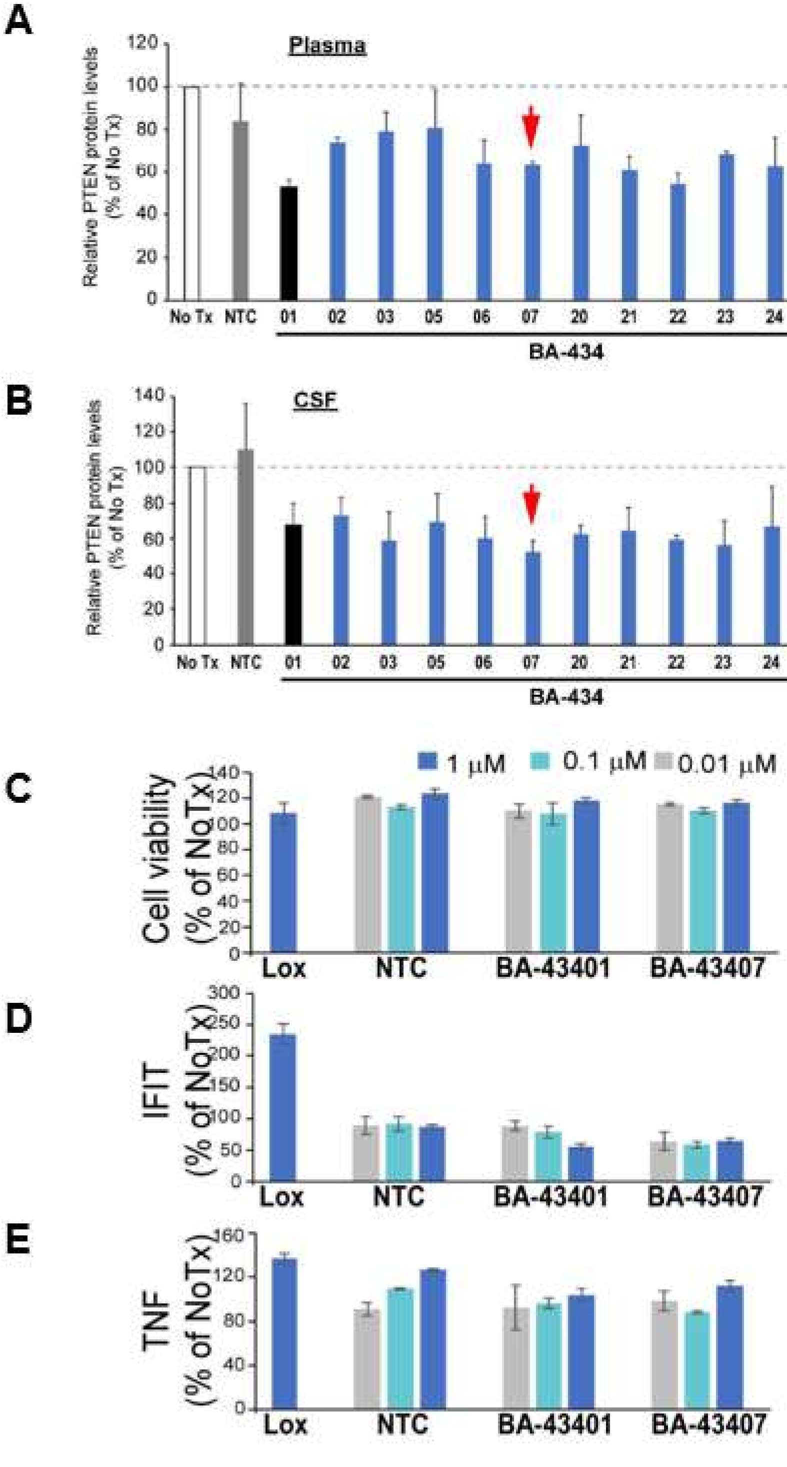

Figure S5. Stability of PTEN sdRNA in biological matrices and absence of immune system stimulation. Related to Figure 5. BA-434 series sdRNAs were incubated in either (A) adult rat plasma or (B) rat cerebrospinal fluid (CSF) for 72 hours before being added to cortical neuron cultures. (C) Primary human peripheral blood mononuclear cells (PBMCs) were treated with the sdRNAs for 24 hours and cell viability determined by Alamar Blue staining. Immunostimulation was evaluated by interferon-induced protein with tetratricopeptide repeats (IFIT) induction (D), or tumor necrosis factor (TNF) induction (E). Loxoribine (Lox) was used as positive control for immune stimulation.

Supplemental Figure 6

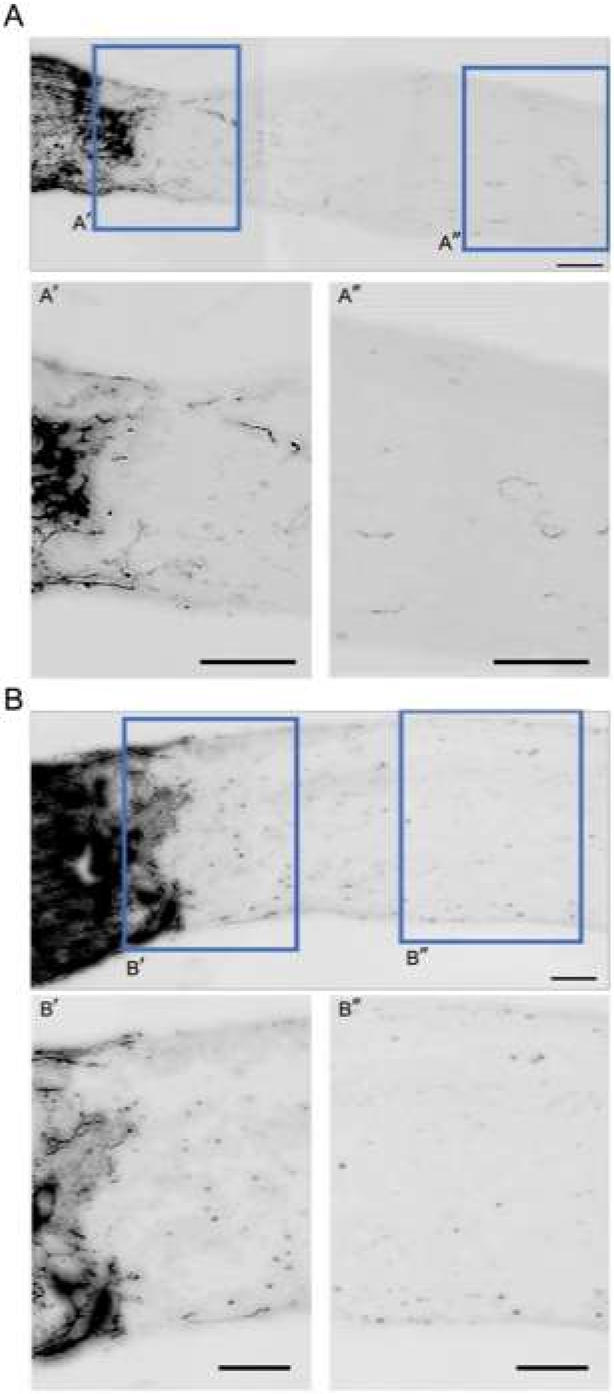

Figure S6. RGC axons do not regenerate in the absence of treatment with sdRNA. Related to Figure 6. (A) Low magnification image showing the optic nerve, and higher magnification views of (A') site adjacent to the crush site and (A'') more distal to the crush site after injection of BA-434-007 into the vitreous. (B) Comparison showing the optic nerve from a sterile PBS-injected optic nerve from one of a group of animals operated on the same day. All optic nerves were anterogradely labeled with fluorescent CTB 24 h hours before fixation and visualization.

ID	Target Sequence
BA-431	TTTTGGATTCAAAGCATAAA
BA-432	ATCTTGACAAAGCAAACAAA
BA-433	AATATTGATGATGTAGTAAG
BA-434	TATTATAGCTACCTGTTAAA
BA-435	ATATTTATCCAAATATTATT
BA-436	TCCTGCAGAAAGACTTGAAG
BA-437	CGTTAGCAGAAACAAAAGGA
BA-438	TTCCTGCAGAAAGACTTGAA
BA-439	GTTCTTCCACAAACAGAACA
BA-440	AACCATTACAAGATATACAA
BA-441	TTATTATAGCTACCTGTTAA
BA-442	GAGTTCTTCCACAAACAGAA
BA-443	CTTTGTGATCAGGAAATCGA
BA-444	AGTAAGGACCAGAGATAAAA
BA-445	CTGTGTGTGGTGACATCAAA
BA-446	TTTGGATTCAAAGCATAAAA
BA-447	AGGTGAAGTTATACTTCACA
BA-448	TCTTCCACAAACAGAACAAG
BA-449	ACATTATGACACCGCCAAAT
BA-450	GACAAAGCAAACAAAGACAA

Table S1. PTEN targeting sequences from primary screen. Sequences common to rat and human PTEN were determined by *in silico* analysis.

Oligo Name	Sequence (5’ to 3’)
GAPDH F1 (rat)	CCCCCAATGTATCCGTTGTG
GAPDH R1 (rat)	TAGCCCATGATGCCCTTTAGT
PTEN F1 (rat)	TAATATACATAGCGCCTCTGACTG
PTEN R1 (rat)	GGACTGGTGTAATGATTTGTGC

Table S2. Oligonucleotides used for qPCR. The primers were used for qPCR of HeLa, PC-12 cells, primary neurons, and adult rat retinal homogenates.

ID	Strand	Sequence & modification pattern
BA434	Sense	5'mU.mA.G.mC.mU.A.mC.mC.mU.G.mU.mU.**A***mA*mA.TEG-Chl
-20	Antisense	5'P.mU.fU.fU.A.A.fC.A.G.mG.fU.A.G.fC.fU***m**A*fU*A***m**A*fU*A
BA434	Sense	5'mU.mA.G.mC.mU.A.mC.mC.mU.G.mU.mU.**A***mA*mA.TEG-Chl
-21	Antisense	5'P.mU.fU.**mU**.A.A.fC.A.G.mG.fU.A.G.fC.fU*A*fU*A*A***mU***A
BA434	Sense	5'mU.mA.G.mC.mU.A.mC.mC.mU.G.mU.mU.**A***mA*mA.TEG-Chl
-22	Antisense	5'P.mU.fU.**mU**.A.**m**A.fC.A.G.mG.fU.A.G.**m**C.fU*A*fU***m**A*A***mU***A
BA434	Sense	5'mU.mA.**m**G.mC.mU.**m**A.mC.mC.mU.**m**G.mU.mU.mA*mA*mA.TEG-Chl
-23	Antisense	5'P.mU.fU.fU.A.A.fC.A.G.mG.fU.A.G.fC.fU***m**A*fU*A***m**A*fU*A
BA434	Sense	5'mU.mA.**m**G.mC.mU.**m**A.mC.mC.mU.**m**G.mU.mU.mA*mA*mA.TEG-Chl
-24	Antisense	5'P.mU.fU.**mU**.A.A.fC.A.G.mG.fU.A.G.fC.fU*A*fU*A*A***mU***A
BA434	Sense	5'mU.mA.**m**G.mC.mU.**m**A.mC.mC.mU.**m**G.mU.mU.mA*mA*mA.TEG-Chl
-25	Antisense	5'P.mU.fU.**mU**.A.**m**A.fC.A.G.mG.fU.A.G.**m**C.fU*A*fU***m**A*A***mU***A

Table S3. **Duplex combinations tested.** To ensure the best potency of PTEN sdRNA, the sense strands that appeared to give the highest efficacy (BA-434-002 and -003) were hybridized with the three most efficacious antisense strand candidates (BA-434-005, -006, and -007). The differences in modification pattern of these new duplexes in comparison to the parent duplex BA-434-001 are highlighted (**bold** nucleotide modification added/changed; **bold** nucleotide modification removed). 2'-O-Methyl modification (m), phosphorothioate modification (*), 2'-fluoro modification (f).
